# Osmoadaptive GLP-1R signalling in hypothalamic neurones inhibits antidiuretic hormone synthesis and release

**DOI:** 10.1016/j.molmet.2023.101692

**Published:** 2023-02-10

**Authors:** Michael P. Greenwood, Mingkwan Greenwood, Soledad Bárez-López, Joe W. Hawkins, Katherine Short, Danijela Tatovic, David Murphy

**Affiliations:** 1 Molecular Neuroendocrinology Research Group, Bristol Medical School: Translational Health Sciences, University of Bristol, Dorothy Hodgkin Building, Bristol, United Kingdom; 2 Diabetes and Endocrinology Department, North Bristol NHS Trust, Bristol, United Kingdom

**Keywords:** Liraglutide, Hypothalamus, Hydration, Vasopressin, Oxytocin, Glucagon like peptide 1

## Abstract

**Objectives:**

The excessive release of the antidiuretic hormone vasopressin is implicated in many diseases including cardiovascular disease, diabetes, obesity, and metabolic syndrome. Once thought to be elevated as a consequence of diseases, data now supports a more causative role. We have previously identified CREB3L1 as a transcription factor that co-ordinates vasopressin synthesis and release in the hypothalamus. The objective here was to identify mechanisms orchestrated by CREB3L1 that co-ordinate vasopressin release.

**Methods:**

We mined *Creb3l1* knockdown SON RNA-seq data to identify downstream target genes. We proceeded to investigate the expression of these genes and associated pathways in the supraoptic nucleus of the hypothalamus in response to physiological and pharmacological stimulation. We used viruses to selectively knockdown gene expression in the supraoptic nucleus and assessed physiological and metabolic parameters. We adopted a phosphoproteomics strategy to investigate mechanisms that facilitate hormone release by the pituitary gland.

**Results:**

We discovered glucagon like peptide 1 receptor (*Glp1r*) as a downstream target gene and found increased expression in stimulated vasopressin neurones. Selective knockdown of supraoptic nucleus *Glp1r*s resulted in decreased food intake and body weight. Treatment with GLP-1R agonist liraglutide decreased vasopressin synthesis and release. Quantitative phosphoproteomics of the pituitary neurointermediate lobe revealed that liraglutide initiates hyperphosphorylation of presynapse active zone proteins that control vasopressin exocytosis.

**Conclusion:**

In summary, we show that GLP-1R signalling inhibits the vasopressin system. Our data advises that hydration status may influence the pharmacodynamics of GLP-1R agonists so should be considered in current therapeutic strategies.

## Highlights

• Identification of *Creb3l1* as a regulator of *Glp1r* expression in the hypothalamus.
• Physiological stimulation of the HNS increases *Glp1r* expression in AVP neurones.
• Knockdown of SON *Glp1rs* decreases food intake, body weight, and HNS outflows.
• GLP-1R agonist liraglutide inhibits hypothalamic *Avp* synthesis.
• Quantitative phosphoproteomics of the pituitary gland reveals possible mechanisms.

## 1 Introduction

There has been a resurgence in interest in the mechanism controlling hypothalamo-neurohypophysial system (HNS) hormone release stemming from clinical associations with obesity and metabolic syndrome [1]. The HNS comprises large magnocellular neurones (MCNs) in the paraventricular nucleus (PVN) and supraoptic nucleus (SON) that make the antidiuretic hormone vasopressin (AVP) and oxytocin (OXT) and release them peripherally into the blood circulation from nerve terminals located in the posterior lobe of the pituitary gland (PP), and within the brain from dendrites and axon collaterals [2]. By signalling at specific G-protein-coupled receptors (GPCRs) in the periphery and centrally, AVP and OXT can regulate physiological processes crucial for the maintenance of homeostasis.

The most recognised roles of AVP in the periphery are the regulation body fluid and cardiovascular homeostasis and the control of blood glucose [3]. OXT is best known for its roles in lactation and parturition and beyond this regulates renal sodium excretion, glucose and insulin homeostasis, gastric motility, eating behaviours, lipid metabolism, and osteogenesis [4]. Interestingly, translational human studies are showing strong therapeutic potential for OXT as a treatment for diabetes and obesity in its own right [4]. There is also increasing experimental, pharmacological, and epidemiological evidence that supports a causative role for sustained increases in circulating AVP in the development of diseases, suggesting the AVP system as a prognostic marker and thus a potential therapeutic target [[5], [6], [7], [8], [9], [10], [11]].

How do feeding signals engage MCNs? When we eat a meal, we ingest food and water, and this has profound effects on osmolality in our body. It has been shown that food intake stimulates the brain which rapidly stimulates thirst to increase water uptake leading to the activation of AVP and OXT MCNs in the SON [12,13]. How does water engage MCNs? A lack of water intake increases plasma osmolality and activates AVP and OXT MCNs in the SON resulting in increased AVP and OXT synthesis and release [14]. The relationship between water intake or hydration status and metabolic health is one that is relatively recent and needs to be better understood. It has been proposed that people who drink less water have a greater chance of developing diabetes and this is related to higher AVP circulating levels [15]. Furthermore, many studies have found that increased AVP release (assessed by measuring the surrogate precursor product copeptin) predicts insulin resistance and the onset of type 2 diabetes and major cardiovascular events in metabolic syndrome patients [[16], [17], [18], [19]]. Thus, it is essential to better understand the signalling mechanism that facilitate HNS hormone release.

We have found that transcription factor CREB3L1 is crucial for regulating hypothalamic AVP and OXT synthesis and secretion [[20], [21], [22], [23], [24], [25]]. We recently showed that CREB3L1 plays a more fundamental cellular role as a regulator of MCN protein synthesis and secretion in order to meet dynamically changing physiological demands [26]. In this study, we have mined our SON *Creb3l1* knockdown (KD) transcriptomic dataset and identified dramatically decreased expression of several GPCRs included the glucagon like peptide 1 receptor (GLP-1R). This receptor is activated by the endogenous ligand glucagon like peptide 1 (GLP-1). There are two major sites of GLP-1 production in our bodies. When food and fluids enter the gut, they stimulate the release of GLP-1 (encoded by the preproglucagon gene, *Gcg*) from intestinal L-cells into the circulation. A separate GLP-1 circuit of neuronal origin originates from the nucleus tractus solitarius (NTS) that targets GLP-1Rs in the brain [27]. GLP-1 is involved in glucose homeostasis via its stimulation of insulin secretion from the pancreas, cardiovascular function, gut motility, and is a physiological satiety factor [27].

The SON receives projections from GLP-1 synthesising neurones in the NTS and these innervate MCNs in the SON [28]. An HNS GLP-1R circuit has been described but a large void remains regarding assessment of function. In this study, we have investigated GLP-1R expression in different models of HNS activation. To determine the physiological role of the MCN GLP-1R population we delivered viruses to KD its expression exclusively in the SON. Using several experimental models both *in vivo* and *ex vivo* including studies with GLP-1R agonist (GLP-1RA) liraglutide, we show that SON GLP-1Rs regulate HNS activity and likely hormone release both basally and following physiological stimulation. By quantitative phosphoproteomics of the pituitary neurointermediate lobe (NIL) after acute liraglutide treatment we have identified hyperphosphorylation events in several proteins located in active zone of the nerve terminal that control hormone release. This new information has the potential to influence current clinical practice with GLP-1RAs.

## 2 Methods

### 2.1 Animals

All experiments were performed under Home Office UK licences 30/3278 and PP9294977 held under, and in strict accordance with, the provisions of the UK Animals (Scientific Procedures) Act (1986); they had also been approved by the University of Bristol Animal Welfare and Ethical Review Board. Animal sample sizes were calculated by making an estimate of variability from previous experiments that we have performed with two groups, using similar approaches, under similar conditions, in rats. These data provide an estimate of the expected standard deviation of the primary outcome and then power calculations in GPower 3.1 have been used to calculate the sample size for the experimental group [29]. In some cases, the same animal was able to serve as its own control with control virus injected into one SON and experimental virus into the other (referred to as SONs per group). All studies were performed with time-matched experimental controls run in parallel and sampled on the same day. For animal studies with two groups, randomisation was performed by the flip of a coin to determine which group the animal was assigned. Viral injections for each animal study were completed in 3 days. In our previous studies injections were missed in approximately 10% of animals so numbers were accordingly increased. Animals to be injected were handled daily.

Male Sprague Dawley rats weighing 250–274 g were purchased from commercial supplier Envigo (RRID: 70508). Rats were housed under a 12:12 light/dark cycle at a temperature of 21–22 °C and a relative humidity of 40–50% with food and water *ad libitum* unless stated. Through the University of Bristol Animal Management Information System, animals are randomly assigned to cages by Animal Services Unit staff. The number of cages, and the number of animals per cage, is pre-set by the investigator on AMIS, but the allocation is independently performed. Rats were housed in groups of 3–4 for a 1–2-week period of acclimation before experimentation. After surgical procedures animals were singly housed (Techniplast, 1290 conventional rat cages). Cages contained sawdust, bedding material, cardboard tubes, and wooden chews for enrichment. In some experimental series rats were placed in metabolic cages (Techniplast) to allow for precise daily measures of food and water intake alongside urine output. Plastic chew toys (discs) were suspended from the cage lid and actively gnawed providing enrichment to their environment. Measures of food, water, and urine were performed by weight. Nighttime measures and injections were performed under dim red lighting.

### 2.2 Activation of the HNS

*Water deprivation (WD)* - For WD protocols drinking water was removed for 1–3 days. In a single study water was returned for 4 h after 3 days of WD.

*Salt loading* - For salt loading protocols drinking water was replaced with 2% (w/v) NaCl in drinking water for 1 or 7 days.

*Hypertonic saline injection* - To acutely activate the HNS a single IP injection of 1.5 ml/100 g body weight of 1.5 M NaCl solution was performed. Animals were randomly allocated into one of six groups, control, 10, 30, 60, 120, and 240 min. After injection animals were placed back in their home cages, and food, but not water, was returned for the duration of the experiment.

*Lactation* - Female rats with litters were purchased from commercial supplier Envigo and acclimatised to the animal facility for 3 days. Animals were killed immediately after the removal of pups (postnatal day 8–9), to ensure a state of lactation was active. Singly housed females without litters were used as controls.

### 2.3 GLP-1RA studies

Liraglutide (Tocris, 6517/1) was prepared in normal saline to a concentration of 100 μg/ml. Injections of 100 μg/kg body weight or normal saline vehicle were performed IP using a 30-gauge insulin syringe (BD). The dose of liraglutide and route of administration has previously been described in the rat [[30], [31], [32]].

#### 2.3.1 GLP-1RA study series 1 – Liraglutide control

Animals were singly housed 3 days before experimentation. Injections were performed during the final 30 min of the light phase before lights off. Animals were killed 2, 4, 12, and 24 h after injection of liraglutide or vehicle. Food and water intake were recorded for the period of study.

#### 2.3.2 GLP-1RA study series 2 - Liraglutide 4-hour WD

Animals were singly housed for 3 days prior to experimentation. Water bottles were removed from cages at +4 h into the dark phase. After 44 h without drinking water, animals were injected with liraglutide or vehicle. Animals were killed 4 h after injection. Food intake was recorded.

#### 2.3.3 GLP-1RA study series 3 – Liraglutide prolonged dehydration

Animals were placed in metabolic cages (Techniplast) to allow for precise daily measures of food and water intake and the collection of urine samples. Animals were acclimatised to metabolic cages for 2 days before basal measures of food and water intake were recorded. The first of a series of injections, liraglutide or vehicle, were performed at the end of the light phase on day 5 and then every 12 h thereafter. This bi-daily delivery regime has been described based on the half-life of liraglutide in the rat [[33], [34], [35]]. Water bottles were removed after the first injection and not returned. Measures of food and urine were performed for the dark and light phases. Urine samples were collected in 1.5 ml tubes and stored at 4 °C. Animals were killed 60 h after the first injection.

#### 2.3.4 Series 4 – Liraglutide NIL phosphoproteomics

To reduce animal variability that can be introduced by the ingestion of food and fluid, water and food were removed approximately 4 h before injection of liraglutide or vehicle and were not returned. Animals were injected with liraglutide or vehicle in the last hour of the light phase. Animals were killed 30 min later and before lights off.

### 2.4 Plasma and hormone measures

Rats were humanely killed by striking the cranium and then immediately decapitated with a small animal guillotine (Harvard Apparatus). Trunk blood was collected in potassium ethylenediamine tetraacetic acid (EDTA)-coated tubes (BD, 368860) and centrifuged at 1600×*g* for 20 min at 4 °C. Plasma for hormone measures was collected in 1 ml aliquots and snap frozen in liquid nitrogen before storage at −80 °C. Brains were rapidly removed from the cranium and placed into a chilled rodent brain matrix (ASI Instruments, RBM-4000c) on ice for separation of the fore and hind brain regions. The brain was placed cut edge down onto aluminium foil resting on pellets of dry ice and immediately covered with powdered dry ice (within 3 min of decapitation). Brains were wrapped in foil and stored at −80 °C. Pituitaries were placed in 1.5 ml tubes containing 0.5 ml of 0.1 M hydrochloric acid and also stored at −80 °C. Plasma and urine osmolality measures were performed by freezing point depression using a Roebling micro-osmometer (Camlab). Plasma glucose concentrations were determined in duplicate by glucose assay (Abcam, ab102517). Plasma hormone measures: Plasma concentrations of copeptin, OXT, and GLP-1 were determined by ELISA (Copeptin, MyBioSource, MBS724037; OXT, Enzo Life Sciences, ADI-901-153 A, RRID:AB_2815012; GLP-1, Sigma-Aldrich, RAB0201). The extraction method for OXT was reverse phase C18 columns (Phenomenex) to capture peptides according to manufacturer's protocols. Samples were eluted and then dried under a gentle flow of nitrogen gas. Samples were reconstituted by vortexing in assay buffer and assayed immediately in duplicate in accordance with manufacturer's protocols. Copeptin and GLP-1 assays were performed in duplicate on whole plasma according to manufactures instructions. Pituitary hormone measures were performed as described previously [26]. The signal was detected on an iMark microplate absorbance reader (Bio-Rad Laboratories).

### 2.5 Cells and treatments

Human Embryonic Kidney cells HEK293T/17 (ATTC, CRL-11268, RRID: CVCL_1926) and Neuro 2a cells N2a (ATTC, CCL-131, RRID: CVCL_0470) were cultured in DMEM (Sigma, D6546) supplemented with 10% (v/v) heat-inactivated foetal bovine serum (Sigma-Aldrich; F9665), 2 mM l-glutamine (Gibco, 25030) and 100 unit/ml of penicillin-streptomycin (Gibco, 15140). Cells were incubated at 37 °C in a humidified incubator with 5% (v/v) CO_2_. Cells were seeded onto tissue culture plates to 60%–70% confluence for experiments.

### 2.6 Luciferase assays

The rat *Glp1r* promoter region −3563 to −1 bp was amplified from rat liver genomic DNA with restriction sites included in primers for restriction digestion and cloning into compatible sites of minimal promoter plasmid pGL4.10 (Promega, E6651). Primers details are found in Supplemental Table 4. Luciferase assays were performed as described previously using Dual Luciferase Reporter Assay System (Promega, E1910) [25].

### 2.7 Virus production and validation

Three *Glp1r* shRNAs were designed using BLOCK-iT™ RNAi Designer (Thermo Fisher Scientific, Supplemental Table 4) and knockdown efficiency was validated in HEK293T/17 cells overexpressing a rat *Glp1r* cDNA. The most efficient shRNA target sequence was GCACGCATGAAGTCATCTTTG with 88.1% KD. *Glp1r* and non-targeting short hairpin RNAs (shRNAs) were cloned into pGFP-A-shAAV (OriGene) as previously described [26]. Adeno-associated virus (AAV) particles (AAV1/2) were produced using a helper free packaging system (Cell Biolabs, VPK-402 and VPK-421) and prepared to titres of 1 × 10^12^ (AAV1/2-shRNA constructs) genome copies per ml in phosphate buffered saline (PBS) for injection as described previously [24]. The production of *Creb3l1* shRNAs has previously been described [24].

### 2.8 Stereotaxic injections of virus into the SON and PVN

Stereotaxic injections were performed as described previously [26]. The success of viral injections was verified at the end of each study by visualisation of the fluorescent reporter in cryostat cut sections or qRT-PCR [26].

### 2.9 Isolation of RNA and qRT-PCR

The collection and processing of brains by punching has previously been described [21,25,26]. For relative quantification of gene expression, the 2^−ΔΔCT^ method was employed [36]. The internal control genes used for these analyses were the housekeeping genes *Rpl19* [[21], [22], [23]] *and Sdha* (NTS). The changes in NTS *Gcg* expression were also found using *Rpl19* as the housekeeping gene. qRT-PCR primer sequences can be found in Supplemental Table 4.

### 2.10 SON and NIL explants

Control and WD rats were killed by striking of the cranium and brains were rapidly removed and placed into a rodent brain matrix (ASI Instruments, RBM-4000C) chilled on ice. A 2 mm coronal brain slice excised between two razor blades and placed onto a 10 cm upturned petri dish chilled on ice. A 2 mm in diameter sample corer (Fine Science Tools, 18035–02) was used to collect SONs. Individual SONs were placed into 500 μl of cold Krebs slice solution (120 mM NaCl, 5 mM KCl, 2.5 mM CaCl_2_, 1.2 mM NaH_2_PO_4_, 1.2 mM MgSO_4_, 25 mM NaHCO_3_, 5.5 mM glucose, pH 7.4) for 1–1.5 h at 4 °C. The osmolality of the slice solution was adjusted using hypertonic saline solution to match the plasma osmolality of control and WD rats to 300 and 315 mOsmol/kgH_2_O, respectively. Individual SONs were moved using a spoon spatula to a 96-well plate containing 250 μl of warm slice solution per well for SONs or 2 ml per well for NILs. One SON for each animal was treated with 100 nM liraglutide (Tocris, 6517/1) and the other vehicle (saline) for 4 h incubated at 37 °C in a humidified incubator with 5% (v/v) CO_2_. NILs were split in half with a sterile scalpel blade and one half was treated with vehicle and the other half with 100 nM liraglutide for 30 min in a 37 °C water bath set at 25 rpm. This concentration of liraglutide is commonly used for *in vitro* studies [37,38]. The SON solution was removed after 4 h and replaced with 200 μl of Qiazol reagent. Tissue samples in Qiazol were transferred to 1.5 ml Biomasher tubes (Takara Bio, Cat No. 9791 A) and disrupted for 15 s with a Biomasher homogeniser. Total RNA was extracted for qRT-PCR. NIL samples were removed from Krebs solution and immediately frozen on dry ice in 0.5 ml tubes.

### 2.11 RNAscope

Frozen brains were sliced into 16 μm (SON) or 20 μm (NTS) coronal sections in a cryostat. A multiplex RNAscope assay was performed using the RNAscope Multiplex Fluorescent Reagent Kit (Advanced Cell Diagnostics, 320850) in accordance with manufacturer's guidelines. RNAscope probes used in this study were purchased from Advanced Cell Diagnostics; Rn-*Glp1r*-C3 (315221-C3), Rn-*Avp*-C2 (401421-C2), Rn-*Gcg*-C2 (315471 C2), Rn-*Oxt* (479631), and Rn-*Fos* (403591). Images were captured using a Leica SP5-II confocal laser scanning microscope attached to a DMI 6000 inverted epifluorescence microscope with Leica acquisition software for SON. Images for data analysis were acquired with a Leica SP5-II AOBS confocal laser scanning microscope attached to a Leica DMI 6000 inverted epifluorescence microscope using a 63× PL APO CS lens with a 3.4-zoom factor. Quantification of *Glp1r* RNA dots in the nucleus (DAPI labelling close to either AVP- or OXT-positive cytoplasm) or cytoplasm (AVP or OXT labelling) of AVP or OXT neurones was performed using a modular workflow plugin for Fiji created by Dr Stephen J Cross from the Wolfson Bioimaging Facility of the University of Bristol, as described [20]. All combinations with *Gcg* were captured using a DMI6000 inverted epifluorescence microscope with Photometrics Prime 95 B sCMOS camera and Leica LAS-X acquisition software.

### 2.12 Immunofluorescence

Rat perfusion and brain processing was performed as described previously [26]. Slices were washed three times for 5 min each in PBS. For antibodies requiring antigen retrieval (AR), sections were incubated in 1 ml of sodium citrate buffer (10 mM, pH6) in 1.5 ml tubes placed in a 90 °C water bath for 30 min. After 20 min cooling at room temperature, sections were removed from AR buffer and washed three times for 5 min each in PBS. Slices were blocked and permeabilised in 3% (w/v) bovine serum albumen (BSA: Sigma-Aldrich, A7906) prepared in 0.3% (v/v) triton-X100/PBS (PBS-T) for 30 min at RT. Primary antibodies were prepared in 1% (w/v) BSA/PBS-T and incubated at 4 °C for 48–72 h. After three washes for 5 min each in PBS, sections were incubated in darkness (covered in aluminium foil) with Alexa Fluor secondary antibodies made in donkey (Thermo Fisher Scientific) prepared in 1% (w/v) BSA/PBS-T for 1 h. Sections were washed three times for 5 min each with PBS with the inclusion of a minute incubation in DAPI (2-(4-amidinophenyl)-6-indolecarbamidine dihydrochloride, 1 μg/ml) prepared in PBS before the final wash. All incubations were performed with gentle rocking in 24-well plates. Slice were mounted onto glass slides with 0.5% (w/v) gelatine (Sigma-Aldrich, G9382) solution and coverslipped with ProLong Gold antifade mounting media (Thermo Fisher Scientific). The primary antibodies used were GLP-1R (1:500; Abcam, ab218532, RRID:AB_2864762), GLP-1 (1:500; Novus Biologicals, NBP2-23558, RRID:AB_2895594), CREB3L1 (1:500; R&D Systems, AF4080, RRID:AB_2086044), neurophysin I (PS41, RRID: AB_2313960) and neurophysin II (PS38, RRID: AB_2315026) (1:200, both kind gifts from Professor Harold Gainer), OXT (1:5000; Peninsula Laboratories, T-5021), FOS (1:25000, Chemicon, Ab-5 (4–17) PC38, RRID:AB_2106755), S100B (1:500, Sigma-Aldrich, S2532, RRID:AB_477499), and tGFP (OriGene, TA150041, RRID:AB_2622256). All combinations with GLP-1R, CREB3L1, GLP-1 required AR. Images were captured using a DMI6000 inverted epifluorescence microscope with Photometrics Prime 95 B sCMOS camera and Leica LAS-X acquisition software. Images being compared were imaged with the same parameters at the same time and were from the same perfusion collection and same immunostaining run as the reference controls.

### 2.13 Western blotting

A 1-mm micropunch (Fine Scientific Tools) was used to collect SON, PVN, organum vasculosum of the lamina terminalis, subfornical organ, area postrema (AP), median preoptic nucleus, arcuate nucleus and nucleus of the solitary tract (NTS) samples from 100 μm coronal sections in a cryostat according to the rat brain atlas by Paxinos and Watson (fifth edition). A 1.5 mm sample core of 1 mm in diameter was collected for the central amygdala. The NIL was dissected from the anterior pituitary at the time of collection and stored at −80 °C. Tubes were removed from dry ice and 80 μl of ice-cold radioimmunoprecipitation assay (RIPA) buffer was added (50 mM Tris-HCl, pH 7.6; 150 mM NaCl, 1% Nonidet P-40 substitute, 0.5% sodium deoxycholate, 0.1% sodium dodecyl sulfate, 1 mM ethylenediaminetetraacetic acid) supplemented with 1 mM protease inhibitor phenylmethylsulfonyl fluoride, protease inhibitor cocktail (Sigma-Aldrich, P8340), and PhosSTOP phosphatase inhibitor cocktail (Roche, 4906845001). Samples were immediately vortexed before being sonicated in tubes kept on iced water for 10 s of sonication (MSE Soniprep 150). Samples were sonicated for 3 rounds of 10 s with intervals on ice of approximately 5 min. Samples were maintained on ice for an additional 30 min, vortexing every 10 min. To remove cellular debris samples were centrifuged at 10000×*g* for 15 min at 4 °C. The supernatant was removed and stored at −80 °C.

Protein samples were prepared to 1 × Laemmli buffer solution (2% sodium dodecyl sulfate, 10% glycerol, 5% 2-mercaptoethanol, 0.002% bromophenol blue and 0.125 M Tris HCl, pH 6.8). Unless stated samples were not denatured. For semiquantitative analysis of protein levels, 20 μg/lane of total protein (determined in duplicate by Bio-Rad Protein Assay with BSA as standards) was loaded for control and WD samples. To assess GLP-1R expression in different brain structures total protein concentration was determined and 25% of the total protein extract was loaded for each sample, which were collected from a control and WD animal. Proteins were fractionated on 10% sodium dodecyl sulfate polyacrylamide gels and transferred to Immobilon®-P PVDF Membrane (MERCK). For molecular weight estimation one lane on each blot was loaded with Novex Sharp Pre-Stained Protein Standard (Life Technologies, LC5800). Membranes were incubated in 5% Amersham ECL Blocking Agent (RPN2125) in Tris-buffered saline (TBST, 150 mM NaCl; 20 mM Tris-HCl, pH 7.6) with 0.1% Tween 20 for 1 h. All blots were firstly probed for the GLP-1R (1:2000, Abcam, ab218532, RRID:AB_2864762) with the antibody prepared in 1% blocking agent. All other primary antibodies; phosphorylated p44/42 mitogen-activated protein kinase (MAPK) (1:2500; Cell Signaling Technology, 4370 T, RRID:AB_2315112), total p44/42 MAPK (1:1000; Cell Signaling Technology, 4696 S, RRID:AB_390780), β Tubulin (1:10000; Covance, MMS-489 P, RRID:AB_10096105), β Actin (1:10000, Proteintech, 66009-1-Ig, RRID:AB_2687938), SNAP25 (phospho-T138) (1:500, Stratech, ORB163730), SNAP25 (1:1000, Santa Cruz Biotechnology, sc-20038, RRID:AB_628264) were prepared in 5% BSA prepared in TBST. After overnight incubation at 4 °C with gentle rocking, membranes were washed three times for 10 min each with TBST. Membranes were incubated for 1 h with secondary antibodies, anti-rabbit (Sigma-Aldrich, A0545, RRID:AB_257896) or anti-mouse (Sigma-Aldrich, A9044, RRID:AB_258431) conjugated with horseradish peroxidase, prepared in the primary antibody dilution buffer (1:20000) with gentle rocking. Membranes were washed three times for 10 min each with TBST. The signal was detected by chemiluminescence using WESTAR® Supernova HRP Detection Substrate (Geneflow, K1-0068) for the GLP-1R and SuperSignal™ West Dura Extended Duration Substrate for other antibodies (Thermo Fisher Scientific, 34075) using a Syngene G:Box imaging system. The immunoblots were stripped in Restore™ Western Blot Stripping Buffer (Thermo Fisher Scientific) for 15 min after probing for phosphorylated p44/42 MAPK before assessing total p44/42 MAPK abundance. Band intensities were determined using Quantity One (Bio-Rad, Hercules, CA, USA).

### 2.14 Protein extraction for phosphoproteomics

The NIL was separated from the anterior lobe of the pituitary by blunt dissection and frozen in a 0.5 ml tube within 90 s of decapitation on dry ice. Total proteins were extracted in RIPA buffer (50 mM Tris-HCl, pH 7.6; 150 mM NaCl; 0.1% sodium dodecyl sulfate; 0.5% sodium deoxycholate; 1% Nonidet P-40; 1 mM EDTA) supplemented with 1 mM PMSF, pierce protease inhibitor cocktail (Thermo Fisher Scientific, A32963) and phosphatase inhibitor cocktail (Thermo Fisher Scientific, A32957). Lysis buffer (80 μl/sample) was added and samples which were immediately sonicated in tubes kept on iced water for 12 s of sonication (MSE Soniprep 150). Samples were sonicated for 3 rounds of 12 s with intervals on ice of approximately 5 min. Samples we maintained on ice for an additional 30 min, vortexing every 5 min. To remove cellular debris samples were centrifuged at 10000×*g* for 20 min at 4 °C. The supernatant was removed and stored at −80 °C. Protein concentrations were determined in triplicate by Bradford assay with BSA standards using an iMark microplate absorbance reader (Bio-Rad Laboratories).

### 2.15 TMT labelling and phosphopeptide enrichment

Aliquots of 100 μg of each sample were digested with trypsin (2.5 μg trypsin per 100 μg protein; 37 °C, overnight), labelled with Tandem Mass Tag (TMTpro) sixteen plex reagents according to the manufacturer's protocol (Thermo Fisher Scientific, Loughborough, LE11 5RG, UK) and the labelled samples pooled. For the phospho proteome analysis, the TMT-labelled pooled sample was desalted using a SepPak cartridge (Waters, Milford, Massachusetts, USA). Eluate from the SepPak cartridge was evaporated to dryness and subjected to TiO2-based phosphopeptide enrichment according to the manufacturer's instructions (Pierce). The flow-through and washes from the TiO2-based enrichment were then subjected to FeNTA-based phosphopeptide enrichment according to the manufacturer's instructions (Pierce). The phospho-enriched samples were again evaporated to dryness and then resuspended in 1% formic acid prior to analysis by nano-LC MSMS using an Orbitrap Fusion Lumos mass spectrometer (Thermo Fisher Scientific).

### 2.16 Nano-LC mass spectrometry

Phospho-enriched fractions (Phospho-proteome analysis) were further fractionated using an Ultimate 3000 nano-LC system in line with an Orbitrap Fusion Lumos mass spectrometer (Thermo Scientific). In brief, peptides in 1% (vol/vol) formic acid were injected onto an Acclaim PepMap C18 nano-trap column (Thermo Scientific). After washing with 0.5% (vol/vol) acetonitrile 0.1% (vol/vol) formic acid peptides were resolved on a 250 mm × 75 μm Acclaim PepMap C18 reverse phase analytical column (Thermo Scientific) over a 150 min organic gradient, using 7 gradient segments (1–6% solvent B over 1 min, 6–15% B over 58 min, 15–32%B over 58 min, 32–40% B over 5 min, 40–90%B over 1 min, held at 90% B for 6 min and then reduced to 1% B over 1 min) with a flow rate of 300 nl per minute. Solvent A was 0.1% formic acid and solvent B was aqueous 80% acetonitrile in 0.1% formic acid. Peptides were ionized by nano-electrospray ionization at 2.0 kV using a stainless-steel emitter with an internal diameter of 30 μm (Thermo Fisher Scientific) and a capillary temperature of 300 °C.

All spectra were acquired using an Orbitrap Fusion Lumos mass spectrometer controlled by Xcalibur 3.0 software (Thermo Fisher Scientific) and operated in data-dependent acquisition mode using an SPS-MS3 workflow. FTMS1 spectra were collected at a resolution of 120000, with an automatic gain control (AGC) target of 200000 and a max injection time of 50 ms. Precursors were filtered with an intensity threshold of 5000, according to charge state (to include charge states 2–7) and with monoisotopic peak determination set to Peptide. Previously interrogated precursors were excluded using a dynamic window (60s +/−10 ppm). The MS2 precursors were isolated with a quadrupole isolation window of 0.7 *m*/*z*. ITMS2 spectra were collected with an AGC target of 10000, max injection time of 70 ms and CID collision energy of 35%. For FTMS3 analysis, the Orbitrap was operated at 50000 resolution with an AGC target of 50000 and a max injection time of 105 ms. Precursors were fragmented by high energy collision dissociation (HCD) at a normalised collision energy of 60% to ensure maximal TMT reporter ion yield. Synchronous Precursor Selection (SPS) was enabled to include up to 10 MS2 fragment ions in the FTMS3 scan.

### 2.17 Data processing

The raw data files were processed and quantified using Proteome Discoverer software v2.1 (Thermo Fisher Scientific) and searched against the UniProt Rat database (downloaded July 2021: 35859 entries) using the SEQUEST HT algorithm. Peptide precursor mass tolerance was set at 10 ppm, and MS/MS tolerance was set at 0.6 Da. Search criteria included oxidation of methionine (+15.995 Da), acetylation of the protein N-terminus (+42.011 Da) and Methionine loss plus acetylation of the protein N-terminus (−89.03 Da) as variable modifications and carbamidomethylation of cysteine (+57.0214) and the addition of the TMTpro mass tag (+304.207) to peptide N-termini and lysine as fixed modifications, phosphorylation of serine, threonine and tyrosine (+79.966) were included as variable modification. Searches were performed with full tryptic digestion and a maximum of 2 missed cleavages were allowed.

### 2.18 Phosphopeptide abundance processing

The phosphorylation status of identified peptide spectral matches (PSMs) was determined by PD2.4 and the site of phosphorylation predicted by PD2.4 using the PhosphoRS module. Phosphorylation sites predicted by PhosphoRS with greater than 70% confidence were taken as the likely phosphorylation site, and phosphopeptides identified with identical sequences and predicted phosphorylation sites, were combined to provide improved quantitation and confidence. The number of PSMs used to calculate the phosphosite abundance is shown in the “Contributing PSMs” column in Supplemental Table 2. Where a peptide is predicted to be phosphorylated (based on its mass), but the software is unable to assign the site, the site is listed as “Ambiguous”. Where multiple phosphorylation events are unable to be located to specific sites within a peptide, the word “Ambiguous” is repeated the corresponding number of times. As peptides can often be matched to multiple proteins, the list of proteins to which each peptide matched was searched against the list of master proteins in the Total Protein analysis, and if a matching protein was identified, this protein was used as the master protein for that peptide.

### 2.19 Statistical analysis

Image brightness and contrast adjustments were made to the whole image in Leica LAS X software and matched for comparisons. Statistical analyses and plotting of data were performed in GraphPad Prism version 9. Statistical differences between two experimental groups were evaluated using independent-sample unpaired t tests. One-way ANOVA with Tukey's or Dunnett's *post hoc* tests were used to determine the difference between more than two samples with only a single influencing factor. Two-way ANOVA with a Šídák *post hoc* test was used to compare control and experimental values at each timepoint in physiological studies. Two-way ANOVA with Tukey's post hoc test was used to compare every mean with every other mean. Statistical analysis of qRT-PCR data was performed using delta Ct values. A Grubbs' test was performed in GraphPad to identify any significant outliers with an Alpha = 0.05. Viral injection misses confirmed by qRT-PCR or through the visualisation of the GFP reporter expression were not included in analyses. Two animals were removed from liraglutide experimental series 4 after 36 h as they did not show the classic decrease in food intake or urine output on day 1 (0–12 h) of liraglutide treatment but did on day 2 (24–36 h). This is likely due to the first injection being missed as documented for the IP injection route [39]. Statistical significance for phosphoproteomics was then determined using Welch's t tests between the conditions of interest. Since it has been discussed that the use of multiple testing corrected false discover rate (FDR) may be too blunt and restrictive for proteomic analysis [18], especially when analysing such a heterogeneous and complex tissue as the brain [[19], [20], [21], [22], [23], [24]], we considered uncorrected p ≤ 0.05 as differentially expressed phosphosites in our NIL analysis. Data are presented as the mean ± SEM where p ≤ 0.05 was considered statistically significant.

### 2.20 Gene ontology and pathways analysis

Transcriptomic gene ontology (GO) and Kyoto Encyclopaedia of Genes and Genomes (KEGG) pathway analyses were performed in ShinyGO v.76 [40]. GO and KEGG analysis for SON *Creb3l1* KD RNA-seq genes with log_2_ fold change less than < -1 were performed by comparing with a background of all expressed genes by RNA-seq filtered for a baseMean >10. Phosphoproteomics gene ontologies were performed using SynGO [41]. In SynGO we used brain expressed genes as the background list and terms were identified as significant if they were enriched at 1% FDR.

### 2.21 Data availability

We mined data from SON *Creb3l1* KD RNA-seq which has been banked in NCBI's Gene Expression Omnibus under SuperSeries GSE200402 (https://www.ncbi.nlm.nih.gov/geo/query/acc.cgi?acc=GSE200402). All proteomic data has been deposited at the ProteomeXchange Consortium via the PRIDE partner repository with dataset identifier PXD037495. Full western blot lane images can be found in (**supplemental file 1**). All novel materials and raw data are available to the community upon request to the corresponding author.

## 3 Results

### 3.1 SON Creb3l1 KD RNA-seq identifies several GPCRs as Creb3l1 target genes

**Figure 1:**
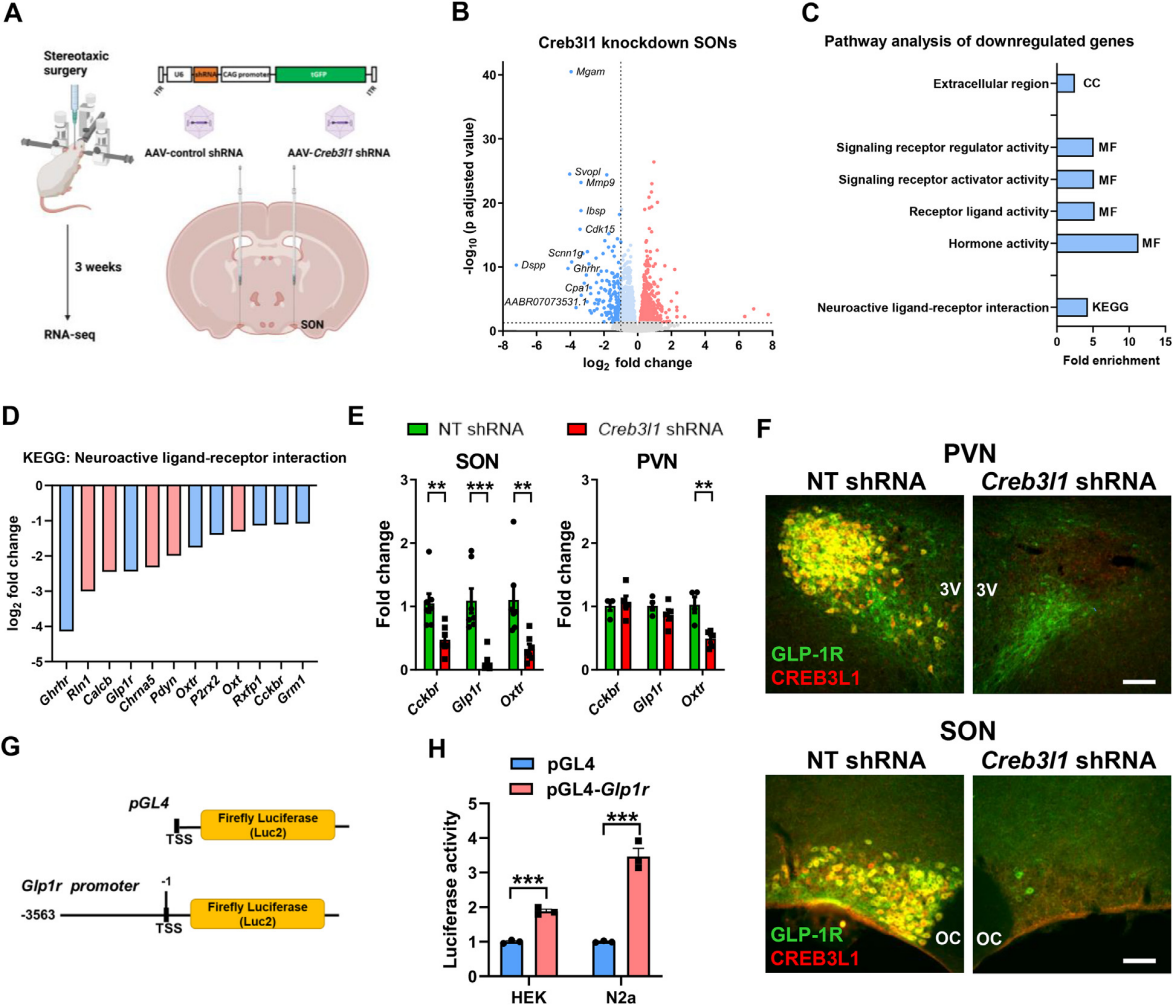
**Identification of the *Glp1r* as a target for regulation by transcription factor CREB3L1. A**, outline of the experimental protocol. AAVs expressing control or *Creb3l1* specific shRNAs were stereotaxically injected into individual SONs of the same animal. SONs were collected for RNA-seq 3 weeks after AAV delivery. B, volcano plot showing differentially expressed genes (DEGs) in *Creb3l1* KD SONs. DEGs are displayed as red (upregulated) and ≤ −1 log_2_ fold change dark blue (downregulated) dots. The top 10 most DEGs genes by log_2_ fold change are displayed. **C**, RNA-seq gene ontology analysis showing enriched terms for Cellular Component (CC), Molecular Function (MF) and Kyoto Encyclopaedia of Genes and Genomes (KEGG) pathways for DEGs ≤ −1 log2 fold change. **D**, bar chart of the gene components of the KEGG pathway Neuroactive ligand–receptor interaction affected by *Creb3l1* KD displayed as log_2_ fold change. GPCRs are shown as blue bars. **E**, relative mRNA expression was investigated by qRT-PCR in the SON and PVN of *Creb3l1* KD animals. **F**, dual immunostaining of CREB3L1 and GLP-1R in control and *Creb3l1* KD PVN and SON of 3-day WD rats. **G**, luciferase reporter vector constructed for the rat *Glp1r* promotor. **H**, luciferase reporter assays were performed in HEK293T and N2a cells in the presence of CREB3L1CA. Luciferase expression was normalised to the expression of the renilla luciferase control reporter vector and to luciferase expression in control treated cells for each cell-line. Values are means + SEM of n = 7 SONs per group, n = 4–5 animals per group (PVN) or n = 3 per group for cell studies. OC, optic chiasm; 3 V, third ventricle. ∗∗p ≤ 0.01, ∗∗∗p ≤ 0.001. Scale bars = 50 μm.

In this study, we asked about downstream gene targets of CREB3L1 in MCNs of the hypothalamus. The experimental protocol for bilateral AAV delivery into SONs is shown in Figure 1A. We focused on genes significantly reduced in expression by ≤ −1 log fold-change in RNA-seq data of *Creb3l1* KD vs. control SONs (Supplemental Table 1 [26]). We show a volcano plot of differentially expressed genes (Figure 1B). To identify gene categories and pathways that might be regulated by CREB3L1, we performed pathway analysis using GO and KEGG (Figure 1C, Supplemental Table 1). A single enriched term in GO: Cellular Component was identified which was Extracellular Region. Four enriched terms were identified in GO: Molecular Function with Hormone Activity and Receptor Ligand Activity being the most significantly enriched. There were no significant GO terms identified for Biological Process. Analysis by KEGG returned a single enriched pathway, KEGG: Neuroactive Ligand–Receptor Interaction. We next asked about downregulated genes in this KEGG pathway. We identified several GPCRs including growth hormone releasing hormone receptor (*Ghrhr*), *Glp1r*, oxytocin receptor (*Oxtr*), and cholecystokinin B receptor (*Cckbr*) (Figure 1D). The RNA-seq basemean for *Ghrhr* was much lower than for other GPCRs so was not investigated further. By qRT-PCR we confirmed significant decreases to *Cckbr* (t = 3.407, p = 0.005), *Glp1r* (t = 6.198, p < 0.001), and *Oxtr* (t = 3.268, p = 0.007) expression in KD SONs (Figure 1E). We further show that *Creb3l1* KD in the PVN decreased *Oxtr* expression (t = 4.615, p = 0.004) (Figure 1E). Of this GPCR trio the *Glp1r was* most strikingly decreased in the SON making it our primary target for further investigation.

To investigate any relationship between GLP-1R and CREB3L1 expression, immunostaining was performed in *Creb3l1* KD animals following water deprivation (WD), a stimulus that robustly increases CREB3L1 expression in AVP MCNs [21]. There was co-expression of CREB3L1 and GLP-1R in PVN and SON MCNs of control virus injected SONs, but expression of both proteins markedly decreased in KD SONs (Figure 1F). Indeed, KD dramatically reduced GLP-1R expression in MCNs of the SON and PVN, but interestingly expression in parvocellular neurones was preserved [42], as was the expression of CREB3L1 in astrocytes [21]. This preserved expression in astrocytes likely reflects the significantly higher tropism of 1/2 viral serotypes for neurones [43]. Further, we have shown that CREB3L1 is expressed predominantly in the MCN component of the PVN [20,21]. This suggested that CREB3L1 regulation of *Glp1r* expression was specific to the MCN cell population, where CREB3L1 is highly expressed and further increases in expression in response to a rise in plasma osmolality [21]. This suggested a novel CREB3L1 mediated *Glp1r* regulatory transcriptional activation pathway in these cells. To test if CREB3L1 could be a transcription factor of the *Glp1r* gene, we created a rat *Glp1r* promoter luciferase construct (Figure 1G). This region of the *Glp1r* promoter contains a consensus CREB3L1 binding site between −2434 and −2429 bp [44]. The overexpression of a constitutively active CREB3L1 mutant (CREB3L1CA [45]) significantly increased promoter activity in HEK293T (t = 13.46, p < 0.001) and N2a (t = 10.62, p < 0.001) cells in agreement with CREB3L1 being a transcriptional regulator of the *Glp1r* gene.

### 3.2 Physiological stimulation of the HNS increases Glp1r expression in AVP neurones

**Figure 2:**
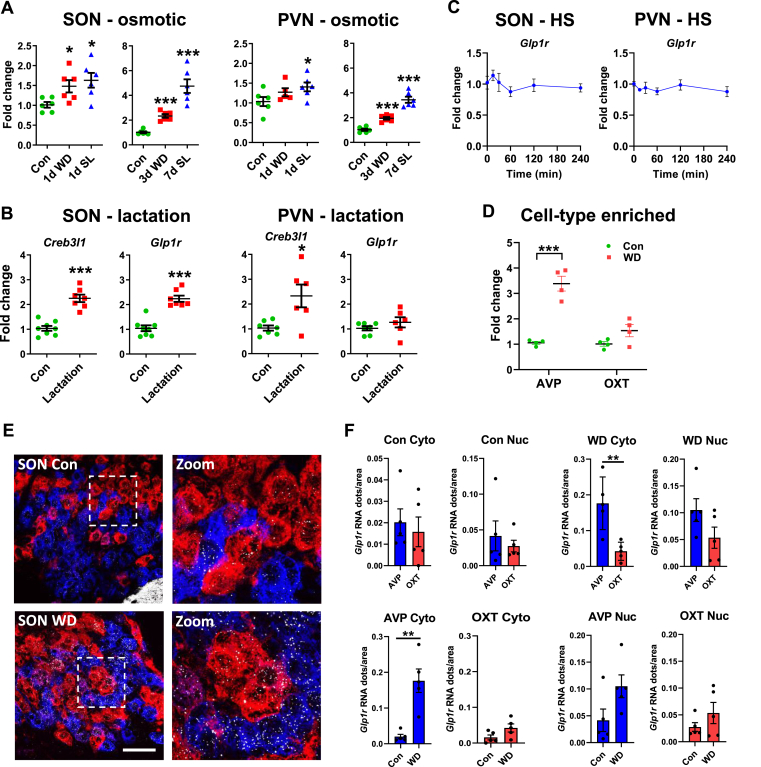
**Activation of the HNS increases *Glp1r* expression in *Avp* MCNs. A**, relative mRNA expression of *Glp1r* was investigated by qRT-PCR in the SON and PVN of 1 day WD and 1 day salt loaded (SL) male rats. **B**, relative mRNA expression of *Creb3l1* and *Glp1r* was investigated by qRT-PCR in the SON and PVN of female control and lactating rats. **C**, relative mRNA expression of *Glp1r* was investigated by qRT-PCR in the SON and PVN of male rats following a single injection of hypertonic saline. **D**, qRT-PCR analysis of *Glp1r* mRNA expression in AVP and OXT punches in control and 3-day WD rat PVN. **E**, fluorescent *in situ* hybridisation to mark the distribution of *Avp* (blue), *Oxt* (red) and *Glp1r* (white) mRNAs in control and 3-day WD SON MCNs. **F**, *Glp1r* RNAscope *in situ* hybridization in control and 3-day WD SONs. Graphs show gene expression as a function of *Glp1r* mRNA dots/cytoplasm (Cyto) or nucleus (Nuc) of AVP and OXT neurones in control and WD conditions. Values are means + SEM of n = 4–7 animals per group. ∗p ≤ 0.05, ∗∗p ≤ 0.01, ∗∗∗p ≤ 0.001. Scale bar = 100 μm.

We next took advantage of archived samples in the lab to investigate PVN and SON *Glp1r* expression in different physiological conditions that elicit increases in AVP and OXT synthesis and release. WD and salt loading (SL) represent classic models to investigate HNS activity [14]. There were significant (one-way ANOVA) alterations to *Glp1r* expression in the SON following acute (F_2,15_ = 5.679, p = 0.015) and chronic (F_2,15_ = 80.05, p < 0.001) osmotic challenges (Figure 2A). Dunnett's multiple comparisons test showed that WD and SL significantly increased *Glp1r* expression (1 d WD, p = 0.040; 1 d SL, p = 0.012; 3 d WD, p < 0.001; 7 d SL, p < 0.001). There were also significant (one-way ANOVA) differences in *Glp1r* expression in the PVN following acute (F_2,14_ = 4.973, p = 0.023) and chronic (F_2,15_ = 73.78, p = p < 0.001) osmotic challenges (Figure 2A). Dunnett's multiple comparisons test showed that WD and SL significantly increased *Glp1r* expression (1 d SL, p = 0.014; 3 d WD, p < 0.001; 7 d SL, p < 0.001). To strengthen a relationship between HNS activity and *Glp1r* expression, we considered PVNs and SONs from lactating rats, a model that increases AVP and OXT release [2,46]. We found significantly higher *Glp1r* expression in SONs of lactating rats (t = 6.466, p < 0.001), but no change in the PVN (Figure 2B). The expression of *Creb3l1* was significantly increased the SON (t = 6.384, p < 0.001) and also the PVN (t = 2.844, p = 0.016) in lactating rats (Figure 2B). In addition, we show that *Glp1r* expression is not increased by a short-term rise in plasma osmolality mediated by acute hypertonic saline injection (Figure 2C).

Our next step was to identify cell populations in the PVN and SON where *Glp1r* is expressed in control SON and following stimulation. We chose WD as the stimulus due to our extensive knowledge of this physiological manipulation. The architecture of the rat PVN allows for enrichment of AVP and OXT neuronal populations by tissue punching [25]. We found increased *Glp1r* expression in AVP enriched punch samples following WD (Figure 2D). In addition, we performed RNAscope to visualise the location of the *Glp1r* mRNA together with *Avp* and *Oxt* in the SON (Figure 2E). Analysis of control SONs showed that the *Glp1r mRNA* is located in cytosolic and nuclear compartments of AVP and OXT MCNs with no difference between cell-types (Figure 2F). However, WD significantly increased *Glp1r* mRNA abundance in the cytoplasm of only AVP MCNs (t = 4.664, p = 0.002). The result of this was significantly more *Glp1r* mRNA in AVP compared to OXT MCNs in WD (t = 3.855, p = 0.005). Thus, we establish that increased *Glp1r* expression in WD is the result of increased expression by AVP MCNs.

### 3.3 WD exclusively increases GLP-1R expression in MCN cell compartments – cell body and nerve terminals

**Figure 3:**
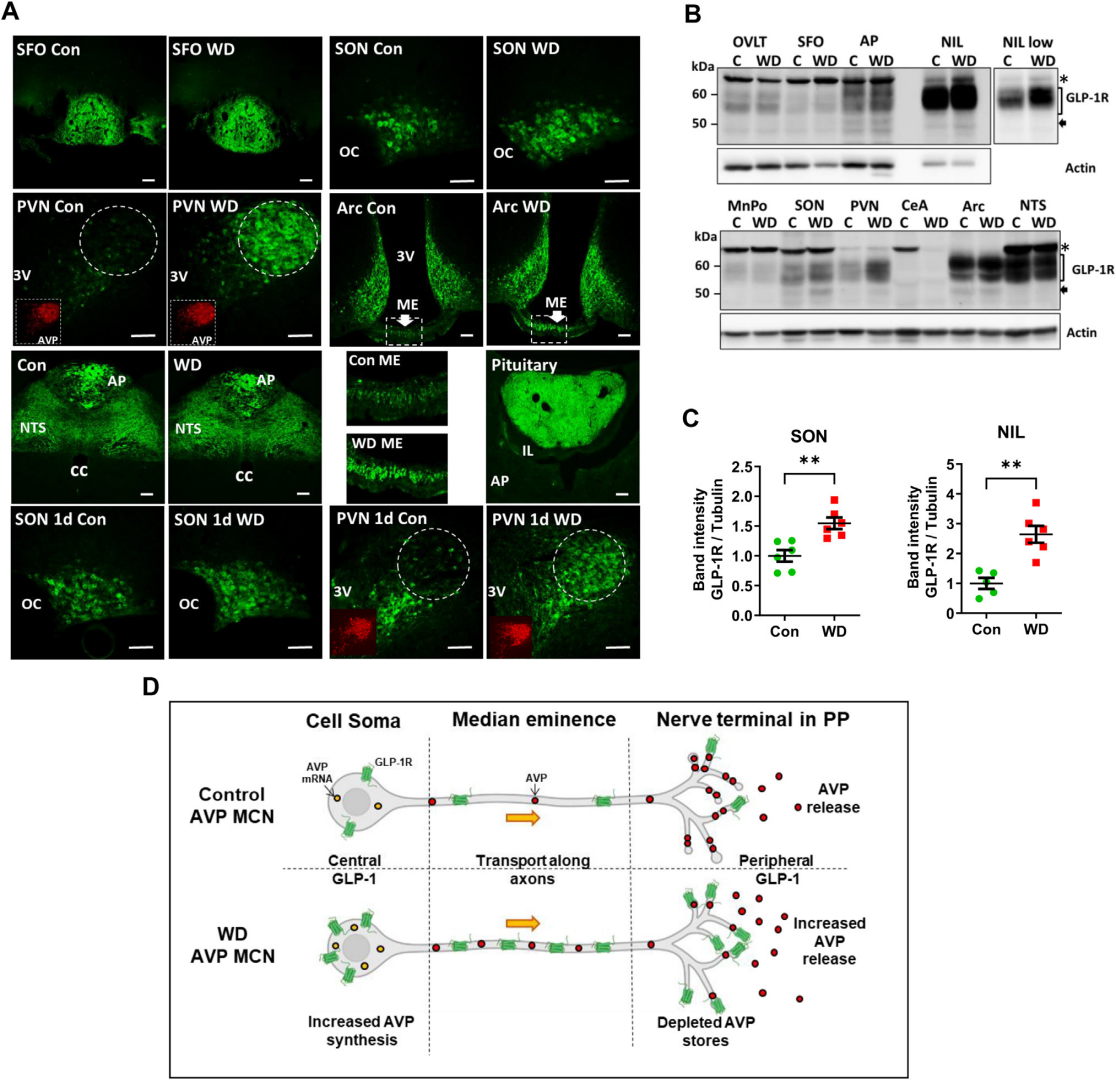
**Discrete changes in GLP-1R expression in the SON and PVN of the WD rat brain. A**, immunostaining of GLP-1Rs in the subfornical organ (SFO), SON, PVN, arcuate nucleus (Arc), NTS, area postrema (AP), median eminence (ME), and NIL of control and 3-day WD rats. Lower 4 panels are for 1 day WD. Inset are panels showing AVP staining (red) in the PVN of control and 1 day and 3-day WD animals. The white broken circle indicates the location of the PVN magnocellular bundle that is highly enriched with AVP expressing MCNs in the rat. The boxed regions of the ME in the Arc images have been expanded to visualise the staining of axons. **B**, western blot of GLP-1R immunoreactive bands in total protein extracts from GLP-1R expressing brain areas from control and 3-day WD rats. The ∗ in this panel indicates non-specific bands confirmed by receptor KD. Other marked bands have been confirmed by receptor KD. A separate image of the NIL captured at lower intensity is shown. Actin is shown as the housekeeping gene. **C**, densitometry analysis of GLP-1R immunoreactive bands in the SON and NIL. **D**, summary diagram of GLP-1R expression in control and WD AVP MCNs. Integrated into this diagram are changes to the synthesis and secretion of AVP in WD. OC, optic chiasm; 3 V, third ventricle; AP, anterior pituitary; IL, intermediate lobe. Values are means + SEM of n = 5–6 animals per group. ∗∗p ≤ 0.01. Scale bars = 50 μm.

We next looked at GLP-1R protein expression in the PVN and SON as well as several other brain regions that express this receptor [42]. It is important to note that we and others have carried out robust and exhaustive validation of the GLP-1R antibody for immunostaining [47,48]. The study of GLP-1R expression in several brain regions suggested that increased expression was unique to AVP MCNs in WD (Figure 3A, Supplemental Figure 1). We further identified alterations to compartments that comprise the HNS, cell soma, median eminence (ME), and posterior pituitary (PP). In addition, we show that GLP-1R expression is increased by 1 day of WD in the PVN and SON. We next performed western blots to validate these observations. Again, it is important to mention that GLP-1R detection by western blotting has been the subject of some debate in the literature. With this in consideration, further antibody controls were carried out looking at N-linked glycosylation of GLP-1R immunoreactive bands (Supplemental Figure 2). The strength of our approach was the application of several techniques to investigate the expression of the GLP-1R at the mRNA and protein levels for a single brain region. We identify GLP-1R immunoreactive bands in the SON and PVN with changes to band intensity consistent with results from *in situ* hybridisation and immunostaining approaches (Figure 3B). The high GLP-1R expression in the arcuate nucleus, NTS, and AP are supported by the literature [42]. GLP-1R immunoreactive bands in the SON (t = 3.921, p = 0.003) and NIL (t = 4.640, p = 0.001) were significantly increased by WD (Figure 3C). The increased molecular weight of the GLP-1R immunoreactive bands is consistent with this receptor being glycosylated [49]. Importantly, there was no detectable expression of the *Glp1r* mRNA in NIL cDNA samples for our previous study as determined here by qRT-PCR [24]. We thus establish increased GLP-1R expression in AVP MCNs in WD (Figure. 3D).

### 3.4 The effects of WD on central and peripheral GLP-1 systems

**Figure 4:**
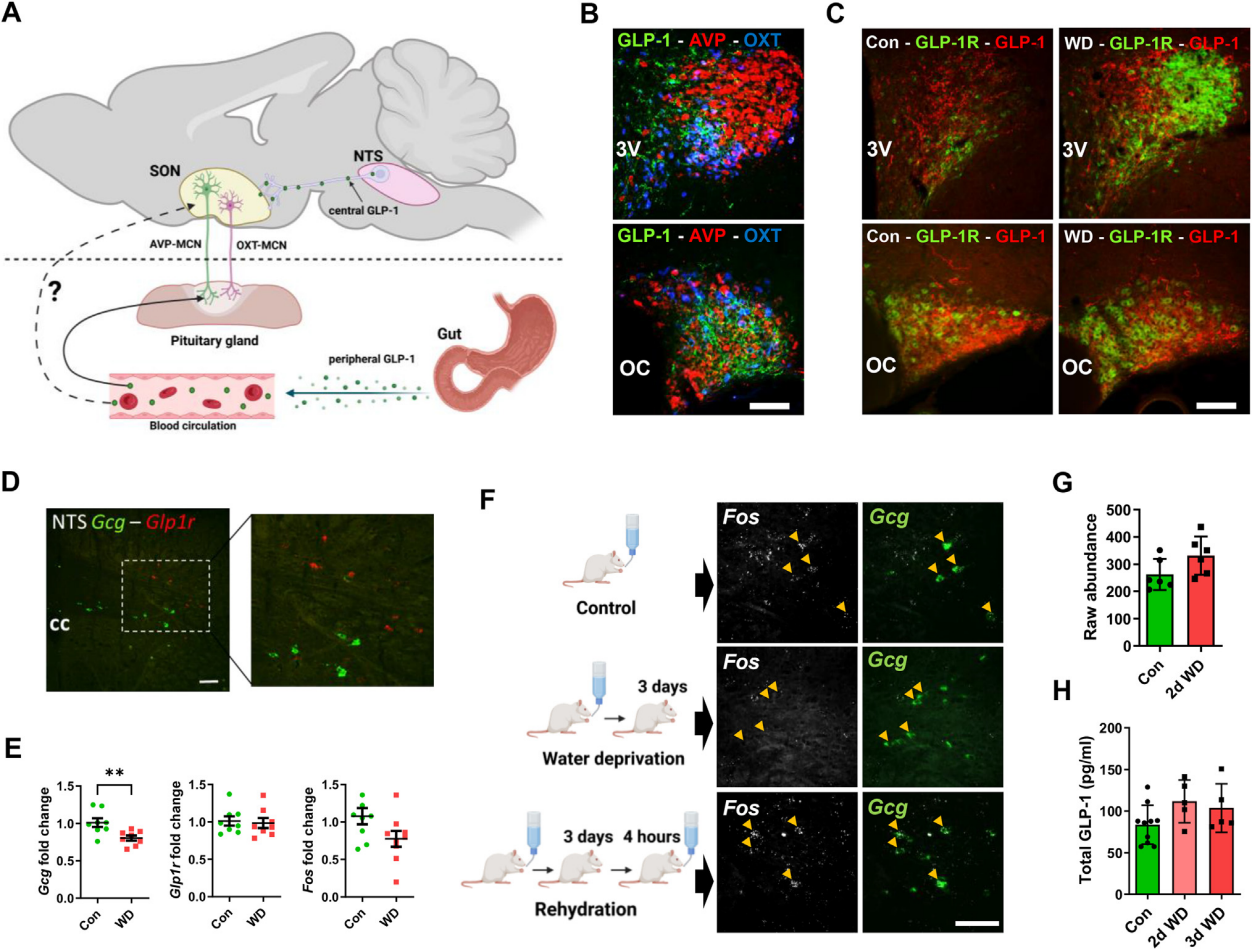
**GLP-1 inputs to the HNS. A**, schematic showing the central (NTS) and peripheral (gut) sources of endogenous GLP-1 capable of signalling the MCN GLP-1R populations. **B**, immunostaining of active GLP-1 containing afferent fibers in the proximity of AVP and OXT neurones in the PVN and SON. **C**, immunostaining of active GLP-1 containing afferent fibres in the proximity of GLP-1R positive neurones in the PVN and SON of control and WD animals. **D**, *in situ* hybridisation of *Gcg* (green) and *Glp1r* (red) expressing cells in the rat NTS. **E**, qRT-PCR analysis of *Gcg*, *Glp1r*, and *Fos* mRNA expression in control and 3-day WD NTS samples. **F**, *in situ* hybridisation of *Fos* (white) and *Gcg* (green) expressing cells in the rat NTS in control, WD, and WD + 4 h rehydration. Arrow heads indicate *Gcg* positive neruones. **G**, total protein raw abundance of GCG in the SON according to LC-MS/MS between control and 2-day WD rats. **H**, plasma levels of total GLP-1 in samples from 2-day and 3-day WD rats. Values are means + SEM of n = 5–8 animals per group. OC, optic chiasm; 3 V, third ventricle; cc, central canal. ∗∗p ≤ 0.01. Scale bars = 100 μm.

We next asked about the effects of WD on central and peripheral GLP-1 systems that likely target HNS GLP-1Rs as shown in Figure 4A. We first asked about active GLP-1 expression in afferent fibers in the PVN and SON using a validated antibody [48]. Immunoreactive GLP-1 positive fibers were found in close proximity to MCNs (Figure 4B) as previously described [28]. The expression and distribution of GLP-1 positive fibers was similar in control and WD rats whilst receptor was increased (Figure 4C). We next moved to determine the location of *Gcg* neurones in the rat NTS by *in situ* hybridization (Figure 4D). In agreement with reports by others [50], *Glp1r* expression was found in a separate population of cells to *Gcg* in the NTS. We found by qRT-PCR that WD significantly decreased *Gcg* mRNA expression in the NTS (t = 3.101, p = 0.010) whilst *Glp1r* and *Fos* expression was not altered (Figure 4E). It is known that WD increases FOS expression by some neurones in the NTS [51]. We asked if *Fos* increased in *Gcg* neurones. We show that WD does not increase *Fos* expression in *Gcg* neurones in the NTS compared to control and rehydration, rather expression appeared to decrease (Figure 4F). In our recent SON proteomics study GCG abundance was similarly not altered by WD ([52], Figure 4G). This is consistent with the decrease to *Gcg* expression. As GLP-1Rs located in the PP possibly receive inputs from circulating GLP-1, we measured total plasma GLP-1 in rats WD for 2 and 3-days (Figure 4H). Measures of total plasma GLP-1 were performed as blood samples were not collected in the presence of dipeptidyl peptidase IV. In the periphery McKay et al. [53] reported no change in gut *Gcg* expression or total plasma GLP-1 in 1-day WD rats consistent with our findings with more prolonged WD. Thus, central and peripheral GLP-1 inputs to the HNS appear not to be significantly altered by WD.

### 3.5 Important physiological roles for the SON population of GLP-1Rs

**Figure 5:**
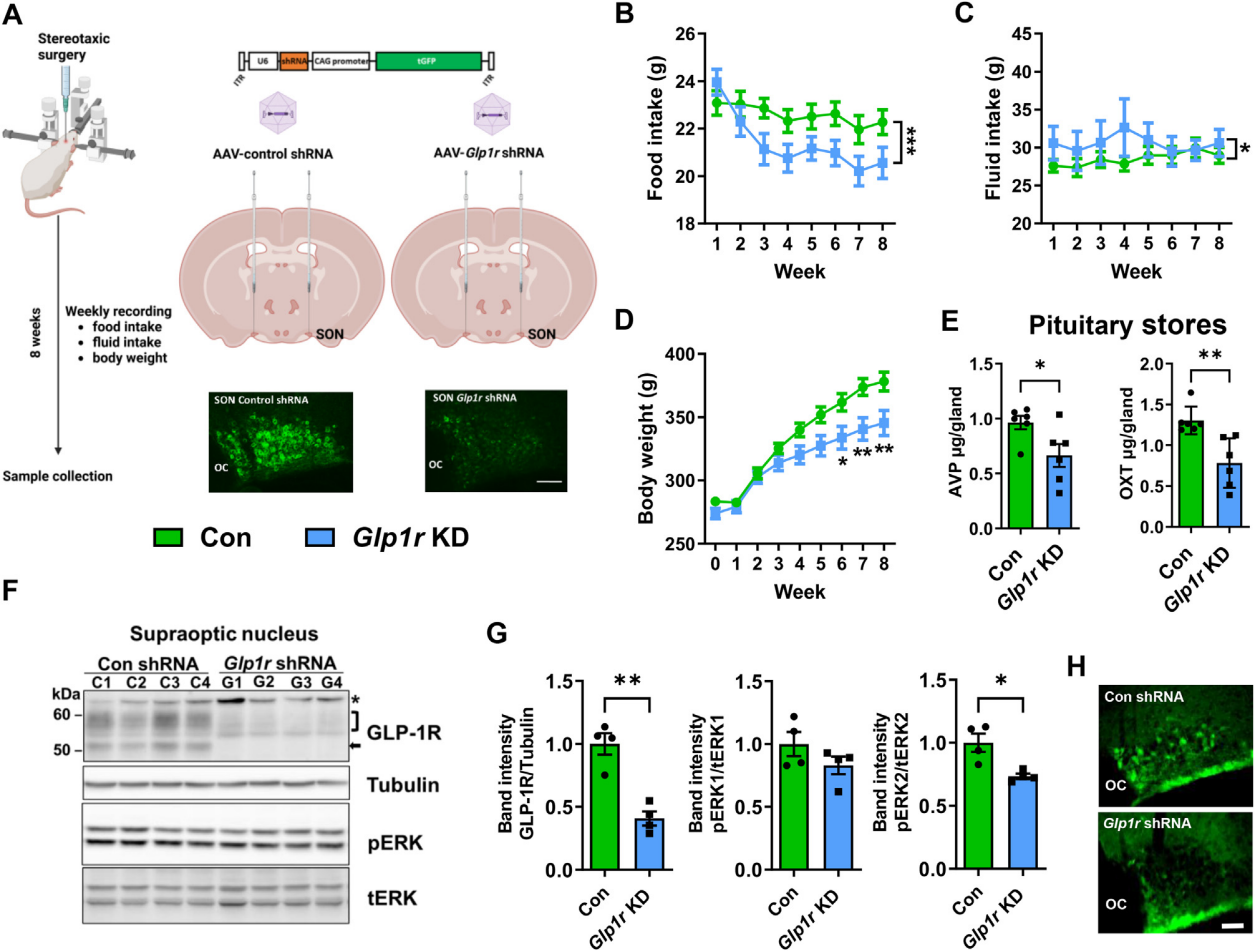
**Assessment of *Glp1r* KD in the SON reveals changes to ingestive behaviour, MCN signalling pathways, and HNS outflows. A**, outline of the experimental protocol involving viral vector deliver of specific shRNAs to KD *Glp1r* expression bilaterally in the SON. The success of gene KD was confirmed by diminished GLP-1R immunostaining in the SON. Food (**B**) and water intake (**C**) and body weight (**D**) were monitored for 8 weeks after viral delivery. **E**, endpoint measures of AVP and OXT pituitary stores determined by ELISA in control and KD animals. **F**, immunoblots of GLP-1R, Tubulin, pERK and tERK immunoreactive bands in SON total protein extracts from control and *Glp1r* KD SONs. **G**, densitometry analysis of immunoreactive bands from F. **H**, immunostaining for pERK in the SON of control and *Glp1r* KD rats. Values are means + SEM of n = 5–8 animals per group. OC, optic chiasm. ∗p ≤ 0.05, ∗∗p ≤ 0.01, ∗∗∗p ≤ 0.001. Scale bars = 50 μm.

To investigate MCN GLP-1Rs functions we used AAVs to deliver *Glp1r* specific shRNAs to rat SONs for gene KD and non-targeting shRNAs as controls (Figure 5A). Importantly, this represents the first study to selectively target SON *Glp1rs* to investigate *in vivo* functions. We measured food intake (Figure 5B), fluid intake (Figure 5C) and body weight (Figure 5D). A two-way ANOVA revealed that KD (viral treatment) significantly altered food (F_1,60_ = 18.76, p < 0.001), water intake (F_1,60_ = 4.473, p = 0.036) and body weight (F_1,180_ = 36.91, p < 0.001). Sidak's multiple comparisons found that KD significantly altered body weight from week 6 compared to control (wk6, p = 0.021; wk7, p = 0.003; wk8, p = 0.003). We and others have shown that peptide stores in the pituitary gland are a reliable metric for determining changes to synthesis/secretion throughout experimental protocols [26,[54], [55], [56]]. We found depleted stores of AVP (t = 2.476, p = 0.033) and OXT (t = 3.683, p = 0.004) in KD rats (Figure 5E). This likely indicates increased hormone release. However, end point measures of plasma copeptin and OXT were not altered (copeptin, con = 73.9 ± 5.9 pg/ml, *Glp1r* = 73.6 ± 3.8 pg/ml; OXT, con 8.97 ± 3.74, *Glp1r* = 7.47 ± 1.97). We next asked about signalling pathways altered in KD SONs. The phosphorylation of (ERK)-1/2 is a pathway that is activated by GLP-1R signalling in the brain [57] as well as being activated in the SON and NIL by WD (Supplemental Figure 3 (30)). To confirm KD we probed blots for GLP-1R expression (Figure 5F). Immunoreactive GLP-1R bands in the SON were significantly diminished in KD samples (t = 5.811, p = 0.001). Moreover, *Glp1r* KD significantly decreased ERK-2 phosphorylation (t = 3.542, p = 0.012) compared to controls (Figure 5G). Immunostaining for phosphorylated ERK1/2 in KD SONs showed diminished expression in MCNs in the SON (Figure 5H). Further, investigation of phosphorylated ERK1/2 in PP nerve terminals of a KD SON also suggested a decrease in ERK1/2 phosphorylation in the pituitary (Supplemental Figure 4). Thus, GLP-1R signalling by ERK1/2 provides a potential signalling pathway to instruct hormone release from soma and dendrites and PP nerve terminals. Therefore, we establish important roles for SON GLP-1 signalling in the regulation of ingestive behaviour which is likely mediated by altered HNS hormone release.

### 3.6 GLP-1RA liraglutide inhibits Avp synthesis both basally and in response to WD

**Figure 6:**
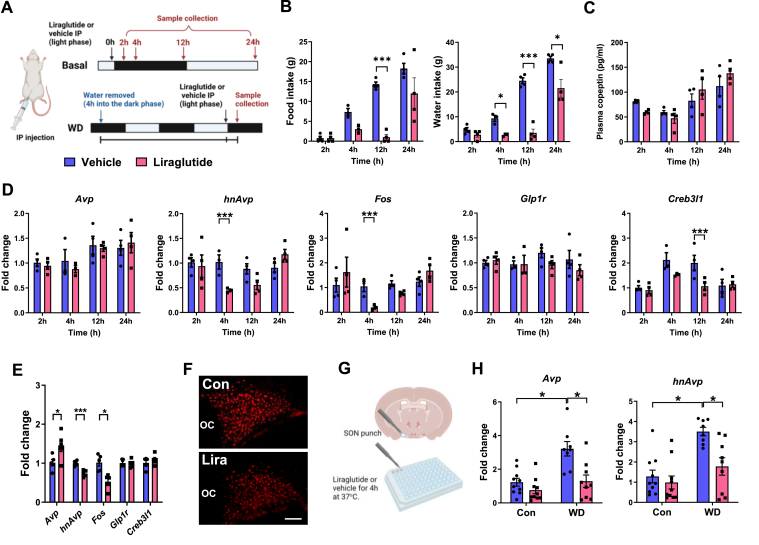
**GLP-1RA liraglutide inhibits *Avp* synthesis and *Fos* expression in the SONs of control and WD rats. A**, outlines of the experimental protocols for liraglutide or vehicle treatment basally and during WD. **B**, Endpoint measures of food intake and water intake. **C**, plasma copeptin 2, 4, 12, and 24 h after a IP injection of liraglutide compared to time-matched vehicle injected controls. **D**, Relative mRNA expression of *Avp*, *hnAvp*, *Fos*, *Glp1r*, and *Creb3l1* in the SONs of rats 2, 4, 12, and 24 h after IP injection of liraglutide compared to time-matched vehicle injected controls. The data has been normalised to the 2-hour vehicle control group. **E**, relative mRNA expression of *Avp*, *hnAvp*, *Fos, Glp1r*, and *Creb3l1* in the SONs of WD rats 4 h after a single IP injection of liraglutide compared to time-matched vehicle injected controls. **F**, immunostaining of FOS expression in the SON of WD rats 4 h after a single intraperitoneal injection of liraglutide compared to time-matched vehicle injected controls. **G**, graphical representation of SON explant isolation for *ex vivo* experimentation. **H**, relative mRNA expression of *Avp* and *hnAvp* in SON explants isolated from control and 3-day WD rats treated with vehicle or liraglutide in the media for 4 h. The data has been normalised to the 4-hour vehicle control group. Values are means + SEM of n = 3–5 animals per group or n = 8–10 SONs per group. OC, optic chiasm. ∗p ≤ 0.05, ∗∗∗p ≤ 0.001. Scale bars = 50 μm.

We next asked about the effects of GLP-1R activation in control and WD states (Figure 6A). In rodents, GLP-1RA liraglutide inhibits food and water intake [53] as we show here in Figure 6B. A two-way ANOVA showed that liraglutide treatment had a significant effect on food (F_1,22_ = 32.13, p < 0.001) and water intake (F_1,22_ = 59.99, p < 0.001). Sidak's multiple comparisons found that liraglutide significantly altered food intake at 12 h (p < 0.001) and water intake after 4 (p = 0.025), 12 (p < 0.001) and 24 (p = 0.030) hours. We next looked at plasma copeptin levels (Figure 6C). A two-way ANOVA showed that liraglutide treatment did not significantly change plasma copeptin. It is worth mentioning that plasma copeptin levels were significantly reduced as determined by t-test at the 2-hour timepoint (t = 8.694, p < 0.001). We next looked at gene expression in the SON (Figure 6D). A two-way ANOVA showed that *hnAvp*, *Fos, and Creb3l1* expression were significantly altered by liraglutide treatment (*hnAvp*, F_1,22_ = 16.40, p < 0.001; *Fos*, F_1,22_ = 5.915, p = 0.024; *Creb3l1*, F_1,22_ = 5.635, p = 0.027). Sidak's multiple comparisons test showed that liraglutide significantly reduced *hnAvp* (p < 0.001) and *Fos* (p < 0.001) expression at 4 h and *Creb3l1* (p = 0.0153) at 12 h.

The next step was to investigate gene expression responses to liraglutide in WD. We chose 4 h liraglutide treatment based on findings in the control state. We show significantly decreased *hnAvp* (t = 5.332, p < 0.001) and *Fos* expression (t = 3.081, p = 0.015), and in contrast increased *Avp* expression (t = 2.400, p = 0.043), but no change to *Glp1r* or *Creb3l1* expression (Figure 6E). It is well known that WD increases *hnAvp* expression in the SON to meet demands for increased synthesis [21]. This decrease in *hnAvp* is unexpected as WD presents a physiological state that requires increased AVP synthesis and release. We further show decreased FOS expression suggesting a decrease in neuronal activity (Figure 6F). We next asked specifically about the MCN GLP-1R population in this response. To address this, we isolated SONs to remove inputs and treated them with vehicle or liraglutide (Figure 6G). A two-way ANOVA revealed that liraglutide significantly alters *Avp* (F_1,33_ = 12.28, p = 0.001) and *hnAvp* RNA expression (F_1,33_ = 7.758, p = 0.009) (Figure 6H). Tukey's multiple comparisons test showed that SON cultures retained properties of WD SONs in cluture with *Avp* (p = 0.034) and *hnAvp* (p = 0.011) expression being significantly elevated above controls. Moreover, the addition of liraglutide to culture media significantly decreased *Avp* (p = 0.014) and *hnAvp* (p = 0.038) expression in WD SONs. Therefore, we establish that GLP-1R activation inhibits *Avp* synthesis and this may be mediated by direct targeting of MCN GLP-1Rs expressed at the cell soma.

### 3.7 GLP-1RA liraglutide increases PP stores of AVP in WD despite increased renal fluid loss

**Figure 7:**
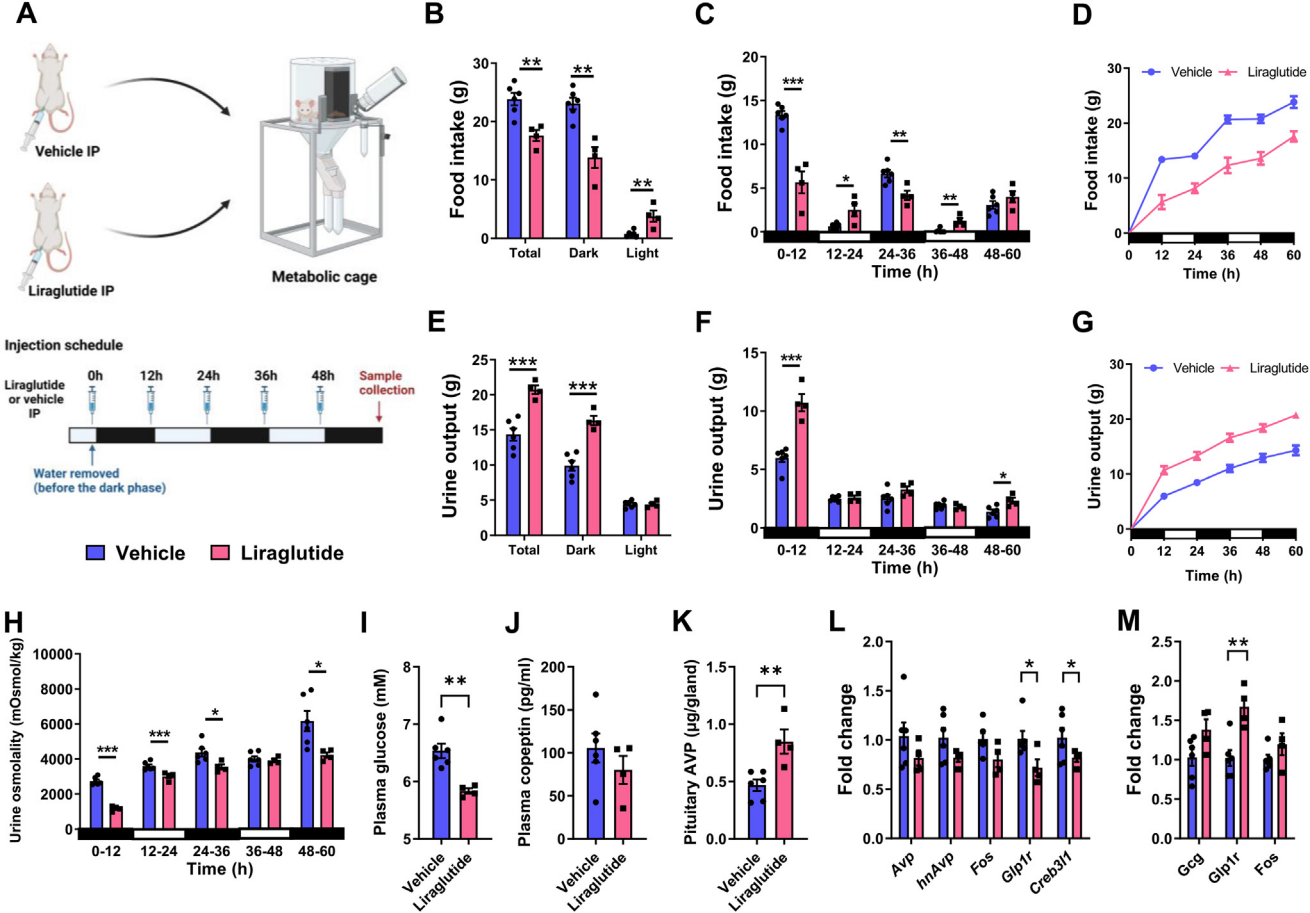
**Liraglutide treatment alters the physiological response to WD. A**, outline of the experimental protocol. Animals were housed in metabolic cages for precise daily measures of food intake and urine output. Animals were injected with liraglutide from the onset and throughout WD. **B**, total food intake during the 60-hour WD protocol and separation of these measures into the dark and light phases of the light cycle. **C**, measures of food intake separated into 12-hour bins corresponding to the dark and light phases of the light cycle. **D**, graph displaying cumulative food intake**. E**, total urine output during the 60-hour WD protocol and separation of these measures into the dark and light phases of the light cycle. **F**, measures of urine output separated into 12-hour bins corresponding to the dark and light phases of the light cycle. **G**, graph displaying cumulative urine output**. H**, measures of urine osmolality separated into 12-hour bins corresponding to the dark and light phases of the light cycle. **I**, endpoint plasma glucose measures in control and liraglutide treatment. **J**, endpoint measures of plasma copeptin in control and liraglutide treated animals measured by ELISA. **K**, AVP pituitary stores were determined by ELISA in control and KD animals. **L,** relative mRNA expression of *Avp*, *hnAvp*, *Fos, Glp1r*, and *Creb3l1* in the SONs in control and liraglutide treated animals. M, relative mRNA expression of *Gcg, Glp1r*, and *Fos* in the NTS in control and liraglutide treated animals. Values are means + SEM of n = 4–6 animals per group. ∗p ≤ 0.05, ∗∗p ≤ 0.01, ∗∗∗p ≤ 0.001.

We next investigated the physiological response to liraglutide in WD rats as outlined in our experimental protocol (Figure 7A). Total food intake was significantly reduced (t = 4.117, p = 0.003) by liraglutide due to significantly decreased (t = 4.933, p = 0.001) dark phase food intake (Figure 7B). In the light phase food intake was significantly increased (t = 3.723, p = 0.006). We analysed daily food intake in 12-hour bins to study dark and light phase feeding patterns (Figure 7C). Liraglutide decreased food intake during the dark phase from 0 to 12 (t = 6.813, p = 0.001) and 24–36 (t = 4.251, p = 0.003) hours, but not 48–60 h. In contrast, liraglutide increased light phase food intake from 12 to 24 (t = 3.067, p = 0.015) and 36–48 (t = 4.251, p = 0.003) hours. The presentation of these data as cumulative food intake showed that liraglutide disrupted feeding and fasting periods (Figure 7D). There was no difference in body weight between the groups (Supplemental Figure 5).

Many renal actions of GLP-1RAs have been described including diuresis and natriuresis [58]. AVP mediated renal water conservation is crucial during WD. We found increased (t = 5.268, p < 0.001) urine output in liraglutide treated animals. This was due to significantly increased (t = 6.321, p < 0.001) urine output during the dark phase (Figure 7E) where urine output significantly increased from 0 to 12 (t = 6.344, p < 0.001) and 48–60 (t = 3.085, p = 0.015) hours (Figure 7F). These data have also been presented as cumulative urine output (Figure 7G). We next asked about urine concentration (Figure 7H). Liraglutide treatment lowered urine osmolality at all timepoints except 36–48 h (0–12, t = 12.28, p < 0.001; 12–24, t = 3.997, p = 0.004; 24–36, t = 2.692, p = 0.0274; 48–60, t = 2.678, p = 0.028). We then asked about plasma parameters. We found significantly lowered plasma glucose levels in liraglutide treated animals (Figure 7I; t = 4.416, p = 0.002). There were no differences in plasma protein (con 44.77 ± 1.31 mg/ml and liraglutide 43.53 ± 0.92 mg/ml), osmolality (con 328.67 ± 1.05 and liraglutide 325 ± 1.10) or copeptin compared to vehicle controls (Figure 7J). However, we found significantly more AVP (t = 3.658, p = 0.006) stored in pituitaries of liraglutide treated animals (Figure 7K). This suggested a reduction in AVP secretion during the course of WD. Interestingly, the expression of *Glp1r* (t = 2.833, p = 0.022) and *Creb3l1* (t = 2.666, p = 0.029) were reduced in SONs of liraglutide treated animals (Figure 7L). In contrast, *Glp1r* expression was increased (t = 4.065, p = 0.004) in the NTS by liraglutide treatment (Figure 7M). Thus, we establish changes to AVP release and altered GLP-1R expression by liraglutide in WD.

### 3.8 NIL phosphoproteomics reveals changes to the phosphorylation of proteins that control vesicle exocytosis from presynaptic nerve terminals

**Figure 8:**
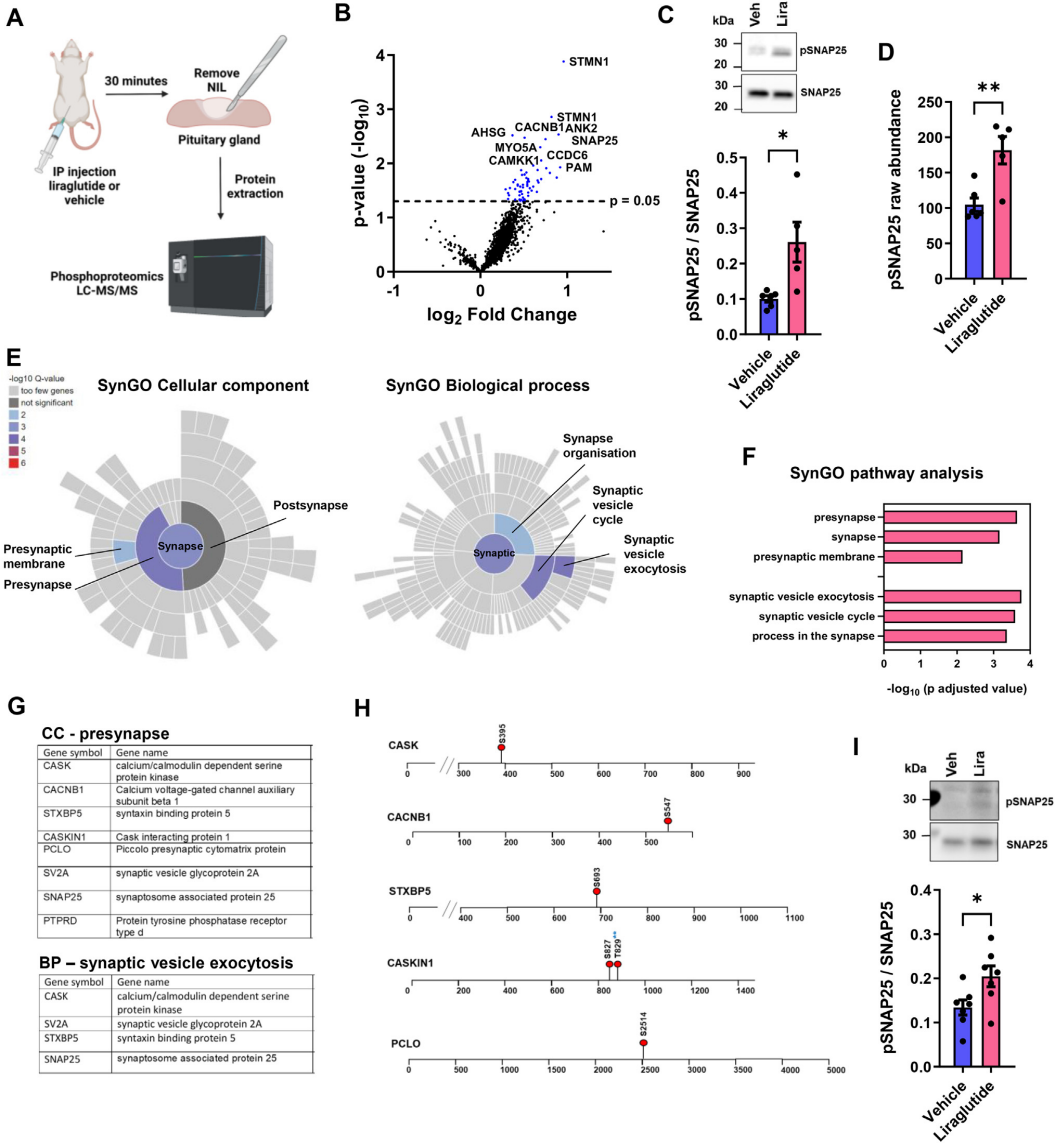
**The liraglutide treated NIL phosphoproteome reveals phosphosite changes to components of the SNARE complex that facilitate vesicular exocytosis. A**, outline of the experimental protocol. **B**, volcano plot of phosphoprotein changes in rat NILs after liraglutide treatment compared to vehicle controls. Selected protein names have been highlighted. **C**, immunoblot of pSNAP25 and SNAP25 immunoreactive bands in the NIL after liraglutide treatment compared to vehicle controls. Densitometry analysis of immunoreactive bands for pSNAP25 and SNAP25. **D**, phospho protein raw abundance of pSNAP25 in the NIL according to LC-MS/MS between control and 30-minute liraglutide treated rats. **E**, analysis of phophoproteomics hyperphosphorylated proteins by SynGO. Figures displaying colour coded enriched terms by Q value for the ontology terms Cellular Component and Biological Process. All level terms identified have been labelled. **F**, bar chart showing SynGO enriched terms for Cellular Component and Biological Process displayed a -log_10_ p adjusted value. **G**, table of hyperphosphorylated proteins containing the overrepresented terms in SynGo ontologies Cellular Component and Biological Process. **H**, mapping of selected phosphosites undergoing hyperphosphorylation in response to 30 min liraglutide treatment compared to vehicle controls. Blues stars indicate the identification of a novel phosphosite for CASKIN1. **I**, immunoblots of pSNAP25 and SNAP25 immunoreactive bands in the NIL after liraglutide treatment *ex vivo* compared to vehicle controls. Densitometry analysis of immunoreactive bands for pSNAP25 and SNAP25 from *ex vivo* NILs. Values are means + SEM of n = 5 animals per group or n = 7 half pituitaries per group. ∗p ≤ 0.05, ∗∗p ≤ 0.01.

Our data so far suggested that activation of GLP-1R leads to the inhibition of AVP synthesis and secretion. To investigate signalling events in nerve terminals of the PP the site of AVP and OXT release, which expresses an abundance of GLP-1Rs [59], we performed a phophoproteomics screen of the NIL after acute liraglutide injection as outlined in our experimental protocol (Figure 8A) The proteomics data can be found in Supplemental Table 2. We identified changes to the phosphorylation status of 45 proteins all of which were hyperphosphorylated (Figure 8B). We validated increased synaptosome associated protein 25 (SNAP25 T138) phosphorylation by western blotting (Figure 8C), in agreement with the phosphoproteomics data (Figure 8D). We next used SynGO to detail synapse specific changes in the NIL (Figure 8E, Supplemental Table 3). We identified three enriched terms in SynGO: Cellular Component with Presynapse being most significantly enriched. We identified three enriched terms in SynGo: Biological Process with Synaptic Vesicle Exocytosis the most significantly enriched. Genes in these terms are tabled in Figure 8F. We next asked about phosphosite changes to other proteins in SynGO terms (Figure 8G). We found hyperphosphorylation events for CASK (S395) a scaffolding protein with a role in synaptic transmembrane protein anchoring and ion channel trafficking, regulator of voltage-gated L-type calcium channels CACNB1 (S547), Stxbp5 (S693) a protein that inhibits membrane fusion between transport vesicles and the plasma membrane, CASKIN1 (S827, T829) a brain-specific adaptor protein for CASK, PCLO (S2514) a scaffold protein of the presynaptic cytomatrix at the active zone which is the place in the synapse where neurotransmitters are released (Figure 8H). We next isolated NILs to remove central and peripheral inputs to focus on signalling by GLP-1Rs expressed in PP nerve terminals. Treatment with liraglutide for 30 min significantly increased (t = 2.430, p = 0.032) SNAP25 phosphorylation at T138 (Figure 8I). Thus, these phosphorylation events may be regulated by directly targeting the nerve terminal GLP-1R population in the PP.

## 4 Discussion

It has been known for decades that receptors for the satiety peptide GLP-1 are abundant throughout the HNS, of rodents and humans [59]. Yet, a large void has remained regarding assessment of their function. Here, we have discovered increased GLP-1R expression in response to physiological stimuli that activate MCNs and increase HNS hormone release. This provided a new and exciting dynamic to assess central and peripheral signaling of the HNS axis by GLP-1 and GLP-1RA liraglutide.

The mechanisms regulating AVP and OXT release have remained rudimentary due to a lack of reliable and precise measurements of circulating levels *in vivo*, with copeptin that is released in an equimolar mode to AVP most used [5]. We, and others, have shown PP stores of AVP and OXT provide a robust measure for changes to secretion, with activation and inhibition of AVP neurones expected to reduce and increase PP stores, respectively [26,[54], [55], [56]]. The KD of SON GLP-1Rs depleted stores of AVP and OXT, and likely increased release. In the 90's, Larsen and colleagues delivered GLP-1 into the rat brain and observed increased AVP release [60], a response reproduced by others [61]. However, Zueco and colleagues reported a decrease in AVP secretion following intravenous administration of GLP-1 in rats [60]. Based on our targeted KD approach and liraglutide treatment, we suggest that activation of MCN GLP-1R population serves to inhibit HNS hormone release.

Several populations of GLP-1Rs in the brain influence feeding behaviour. In this study, changes to feeding are likely the result of altered HNS hormone release. Indeed, central, and peripheral administration of OXT reduce food intake. However, the role of endogenous OXT in feeding behaviours is far more complex and the subject of dispute [62]. Interestingly, vagal afferents express OXT receptors which are required for peripheral OXT-induced eating suppression and represent the major vagal input to NTS GCG neurones [[63], [64], [65]]. This circuit duly provides an OXT feedback mechanism to regulate the activity of MCNs by central GLP-1 circuits innervating the SON. There is also recent evidence that AVP has a role in feeding. The chemogenetic activation of AVP neurones in rats and mice leads to reduction in food intake [66,67]. Therefore, changes to both HNS hormones may contribute to the decrease in food intake in our GLP-1R KD animals as a result of increased release.

The creation of long-acting GLP-1RAs for the treatment of diabetes and obesity has provided a “pharmacological GLP-1 circuit’’ to study GLP-1R functions. When delivered peripherally in rodents long-acting GLP-1RAs including exendin-4, liraglutide, and semaglutide access receptors in the periphery including those in the highly vascularized PP, circumventricular entities of the brain, and within the PVN and SON where MCN cell bodies reside [[68], [69], [70]]. In this study, liraglutide inhibited *Avp* synthesis in control and WD SONs and isolated SONs. This further supported GLP-1 as an inhibitor of HNS activity. We next asked about the physiological effects of liraglutide in WD. Several studies in humans and rodents have shown that GLP-1 and GLP-1RA act on the kidney to induce diuresis and natriuresis [58]. In this study, this response persisted during WD. Our data suggests that decreased AVP release contributes to liraglutide-induced diuresis in the rat. Furthermore, we show that liraglutide decreased plasma glucose levels in WD animals. Liraglutide is known to work by stimulating the secretion of insulin whilst supressing glucagon secretion in a glucose-dependent manner and has been reported not to induce hypoglycaemia [71]. Interestingly AVP is known to stimulate glucagon secretion in rodents and humans and a recent study utilising stimulatory DREADDs to activate SON AVP neurones reported increased circulating copeptin, glucagon and blood glucose levels which were blocked by glucagon receptor and V1bR antagonists [72]. Therefore, the observed decrease in blood glucose may result from reduced AVP release. Thus, we provide a link between the AVP system, hydration status, glucoregulatory health, and liraglutide treatment that may be of clinical importance.

To investigate processes within PP nerve terminals, we performed a phophoproteomics screen of the NIL following acute liraglutide administration. We identified changes to the phosphorylation status of constituents of the SNARE complex that mediates vesicle exocytosis and hormone release [73]. SNAP25 is required for the fusion of synaptic vesicles with the plasma membrane to regulate exocytosis. The phosphorylation of SNAP25 at T138 regulates vesicle priming by inhibiting the assembly of the SNARE complex which is important for neuropeptide release [74,75]. SNAP25 interacts with STXBP5 to regulate AVP secretion in an *in vitro* system and both proteins are highly expressed in PP nerve terminals [76]. Furthermore, synaptic release occurs from a small section of the presynapse nerve terminal called the active zone [77]. Our data shows that liraglutide alters the phosphorylation status of several proteins including PCLO, CASK and CASKIN1 which are scaffold proteins that organise the active zone of the presynapse [78]. In addition, calcium in the PP nerve terminal is crucial for neuropeptide release and the phosphoproteomics data suggests liraglutide induced changes to calcium signalling pathways in the NIL. CASK interacts with N, P/Q, and L-type voltage gated calcium channels as well as several adapter proteins [[79], [80], [81], [82]]. The phosphorylation of L-type calcium channel subunit CACNB1 which regulates channel activity was also increased, and these channels are recognized as part of the molecular machinery for voltage-induced calcium release from internal stores in pituitary nerve terminals [83]. We further identify changes to the phosphorylation of calcium/calmodulin dependent protein kinase kinase 1 (CAMKK1) in the NIL. The hyperphosphorylation of S458 has been shown to decrease CAMMK1 activity and the presence of calcium/calmodulin has been shown to supress the phosphorylation of this site [84,85]. Taken together, these data suggest that peripheral GLP-1 can regulate AVP release by GLP-1R signalling in PP nerve terminals.

In this study there are some limitations. The dual KD of GLP-1Rs in both AVP and OXT MCNs prevents the association of physiological changes to a single cell-type. The anatomical complexity of the HNS which can receive inputs from peripheral and central sources of GLP-1 also means that we do not yet fully understand the contribution of the separate GLP-1 inputs to HNS hormone release. This is something that is difficult to achieve in an intact system. We opted to measure copeptin in this study due to its superior stability to AVP [86]. However, we do not yet know the relationship between circulating AVP and copeptin following liraglutide treatment. It is known that liraglutide alters numerous metabolic parameters as well as causing diuresis and natriuresis which may alter this balance. In humans, non-specific increases in copeptin have been detected in acute settings such as hyponatraemia, suggesting that it may not always be an appropriate surrogate for AVP release [87]. To collect samples for phosphoproteomics we minimised the post-mortem sampling time to preserve phosphorylation events [88]. Thus, we must acknowledge that our samples also contain melanotrophs of the intermediate lobe which is difficult to quickly and cleanly remove from the PP.

## 5 Conclusions

In summary, we provide new insight and understanding into the role a distinct population of GLP-1R expressing neurones in the brain, an area of timely translational importance, as these receptors are drug targets for treatment of metabolic diseases and new combinational therapies are being formulated to improve treatment regimens. Thus, our findings are of direct clinical relevance and may have implications for risk assessment. For example, suboptimal glycaemic control and consequent dehydration are common during intercurrent illness in people with diabetes. Liraglutide is often used in the treatment of type 2 diabetes. If proven to be the case in humans, the effect on GLP-1RA on HNS hormone release could potentially worsen dehydration and should be held off during intercurrent illness. Some of the common side effects of liraglutide including nausea, which incidentally can be caused by altered AVP release [89], as well as vomiting and diarrhoea which require increased AVP release to prevent dehydration and acute kidney injury, with manufacturers and doctors already stipulating the need to maintain fluid intake. This new understanding may help with treatment regimens to alleviate some of the common contraindications of GLP-1RAs. Our findings further suggest that targeting HNS GLP-1Rs to reduce AVP secretion is one mechanism that may prevent the development of diseases associated with sustained increases in AVP release.

## Author contributions

MPG., MG., DT., and DM: Conceptualization. MPG., MG., SBL., and DM: Methodology. MPG., MG., SBL: Formal Analysis. MPG., MG., SBL., JWH., and KS: Investigation. MPG., MG., and SBL: Supervision. MPG: Writing – Original Draft. All authors: Writing – Review & Editing. MPG., MG., and SBL: Data Curation. MPG., DT., and DM: Funding Acquisition. MPG., and DM: Project administration.

## Data Availability

Data will be made available on request.
