# Supplementary material for: Osmoadaptive GLP-1R signalling in hypothalamic neurones inhibits antidiuretic hormone synthesis and release
Source: Mol Metab. 2023 Feb 10;70:101692. doi: 10.1016/j.molmet.2023.101692 (PMC9969259; doi:10.1016/j.molmet.2023.101692)
Supplement: Multimedia component 1 [file mmc1.zip › Supplemental files/Supplemental Figures.pptx]

## Slide 1
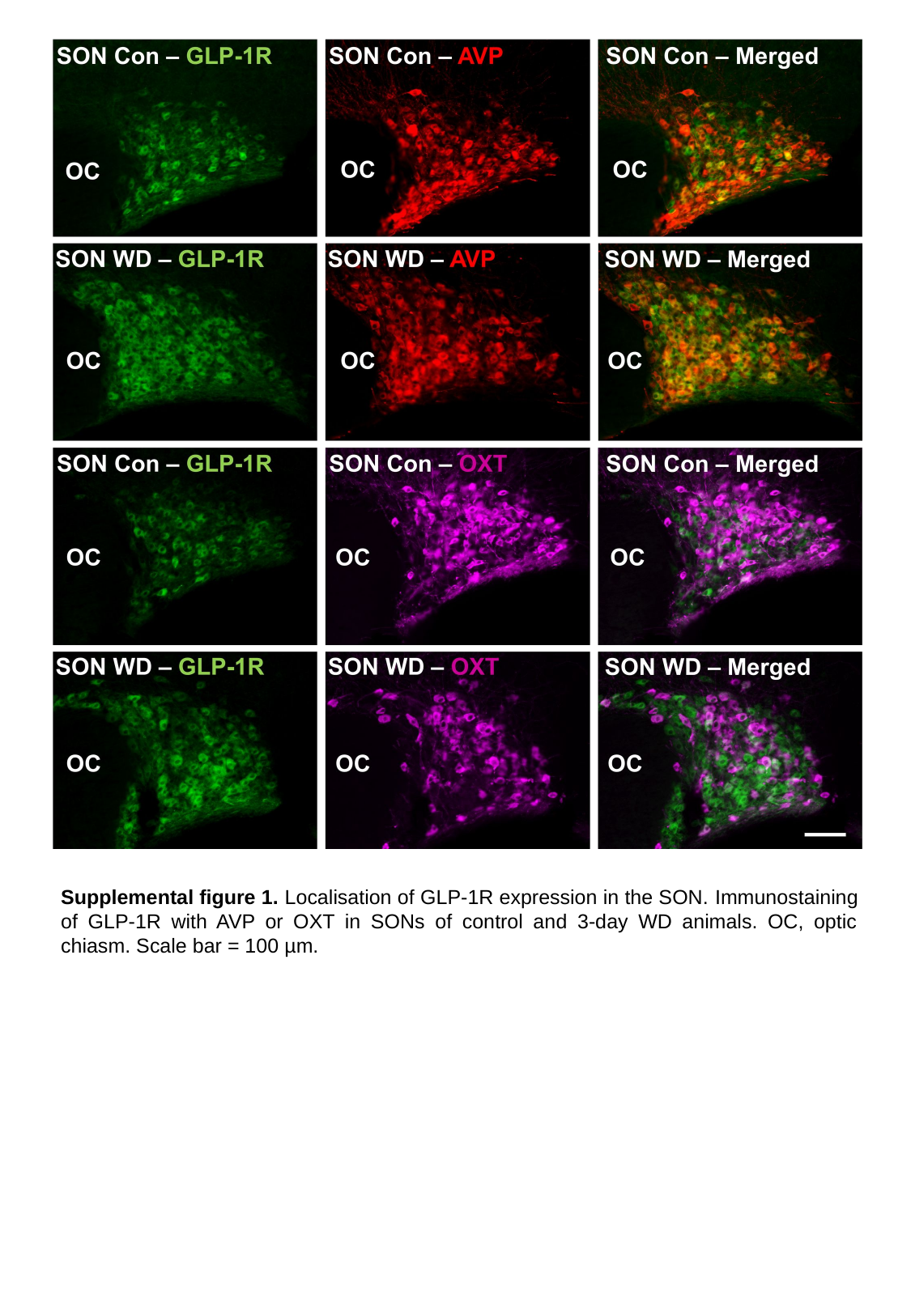

Supplemental figure 1. Localisation of GLP-1R expression in the SON. Immunostaining of GLP-1R with AVP or OXT in SONs of control and 3-day WD animals. OC, optic chiasm. Scale bar = 100 µm.

## Slide 2
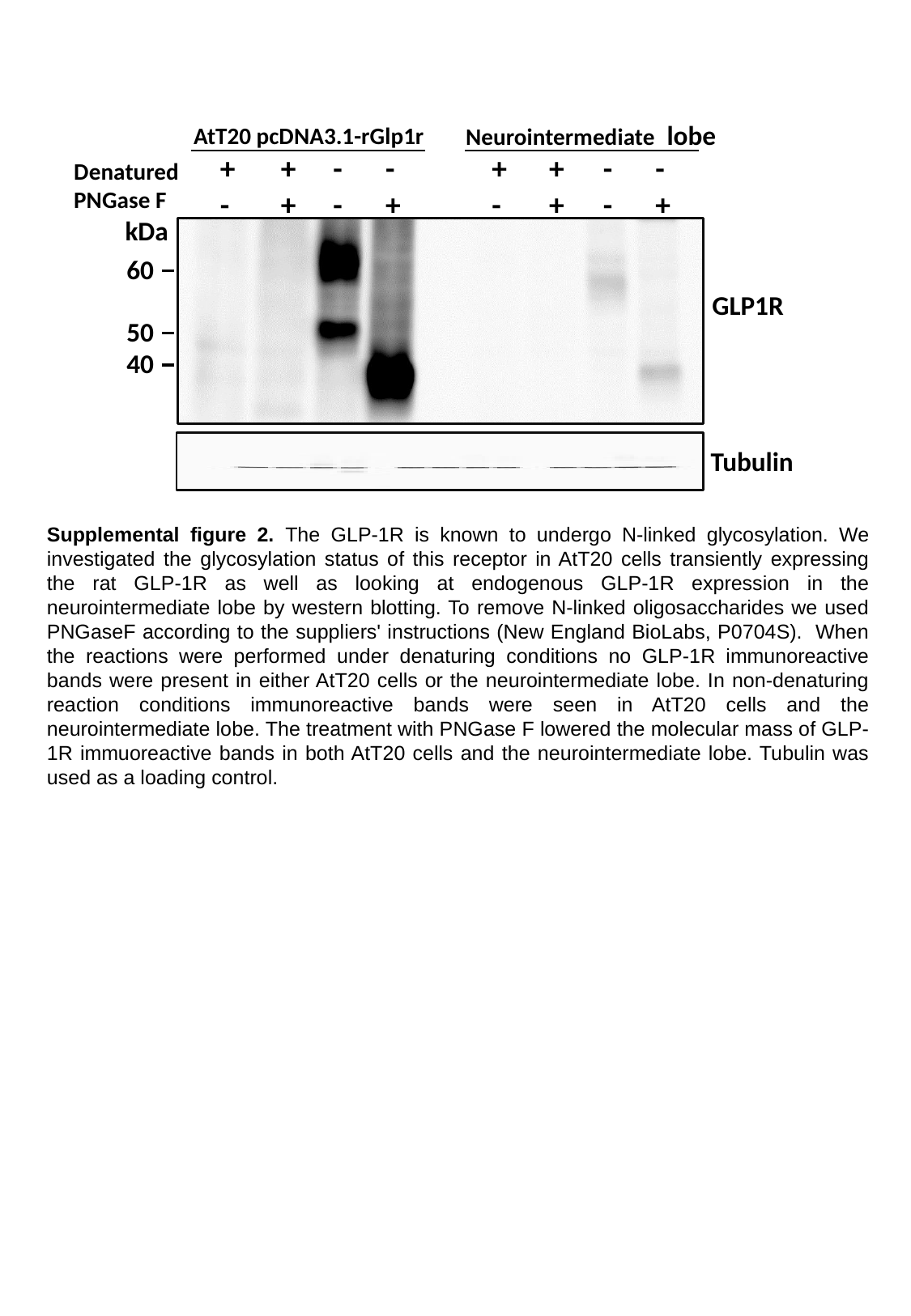

Neurointermediate lobe
AtT20 pcDNA3.1-rGlp1r
+
-
+
+
-
-
-
+
+
-
+
+
-
-
-
+
Denatured
PNGase F
kDa
60
GLP1R
50
40
Tubulin
Supplemental figure 2. The GLP-1R is known to undergo N-linked glycosylation. We investigated the glycosylation status of this receptor in AtT20 cells transiently expressing the rat GLP-1R as well as looking at endogenous GLP-1R expression in the neurointermediate lobe by western blotting. To remove N-linked oligosaccharides we used PNGaseF according to the suppliers' instructions (New England BioLabs, P0704S). When the reactions were performed under denaturing conditions no GLP-1R immunoreactive bands were present in either AtT20 cells or the neurointermediate lobe. In non-denaturing reaction conditions immunoreactive bands were seen in AtT20 cells and the neurointermediate lobe. The treatment with PNGase F lowered the molecular mass of GLP-1R immuoreactive bands in both AtT20 cells and the neurointermediate lobe. Tubulin was used as a loading control.

## Slide 3
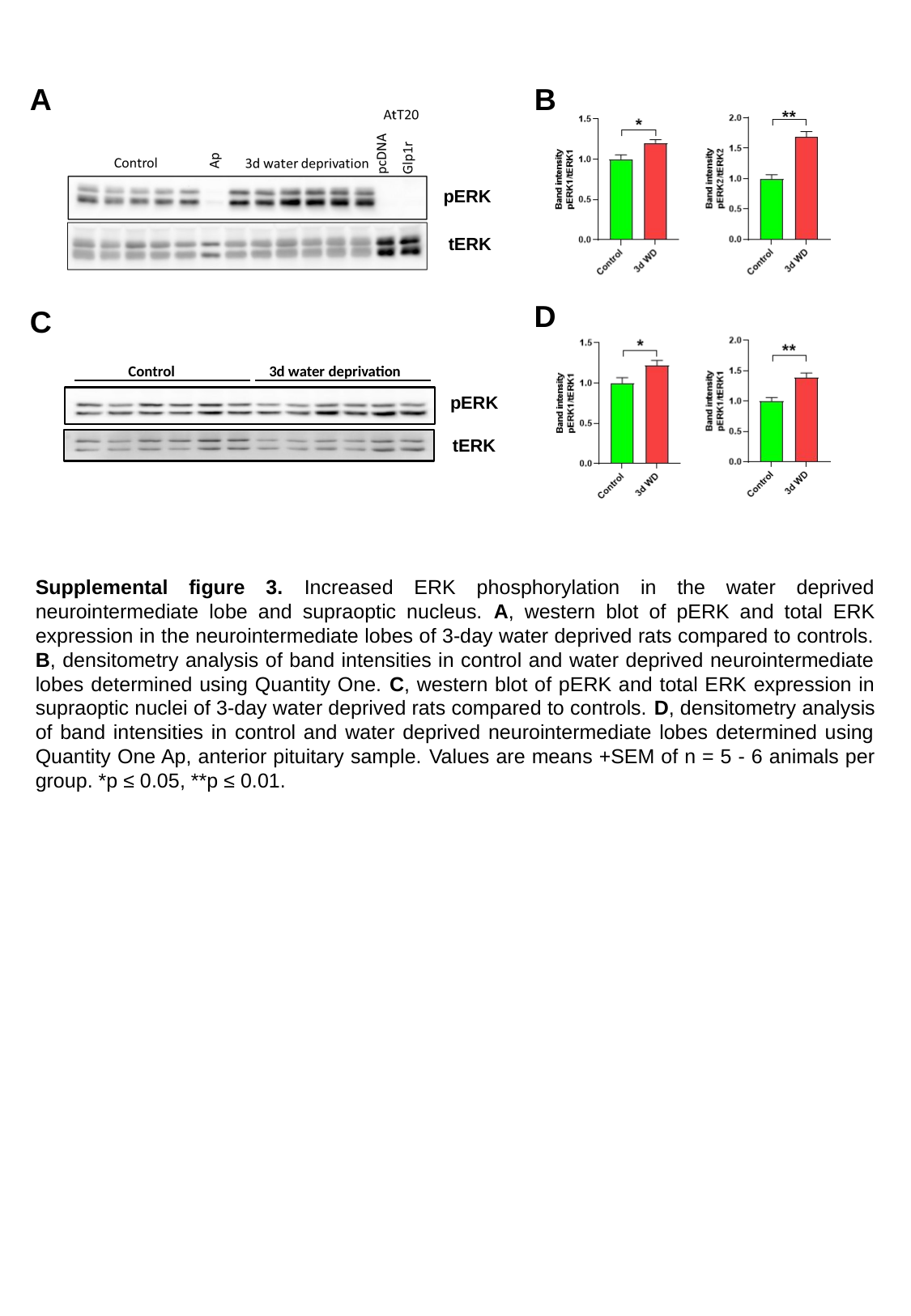

A
B
pERK
tERK
D
C
3d water deprivation
Control
pERK
tERK
Supplemental figure 3. Increased ERK phosphorylation in the water deprived neurointermediate lobe and supraoptic nucleus. A, western blot of pERK and total ERK expression in the neurointermediate lobes of 3-day water deprived rats compared to controls. B, densitometry analysis of band intensities in control and water deprived neurointermediate lobes determined using Quantity One. C, western blot of pERK and total ERK expression in supraoptic nuclei of 3-day water deprived rats compared to controls. D, densitometry analysis of band intensities in control and water deprived neurointermediate lobes determined using Quantity One Ap, anterior pituitary sample. Values are means +SEM of n = 5 - 6 animals per group. *p ≤ 0.05, **p ≤ 0.01.

## Slide 4
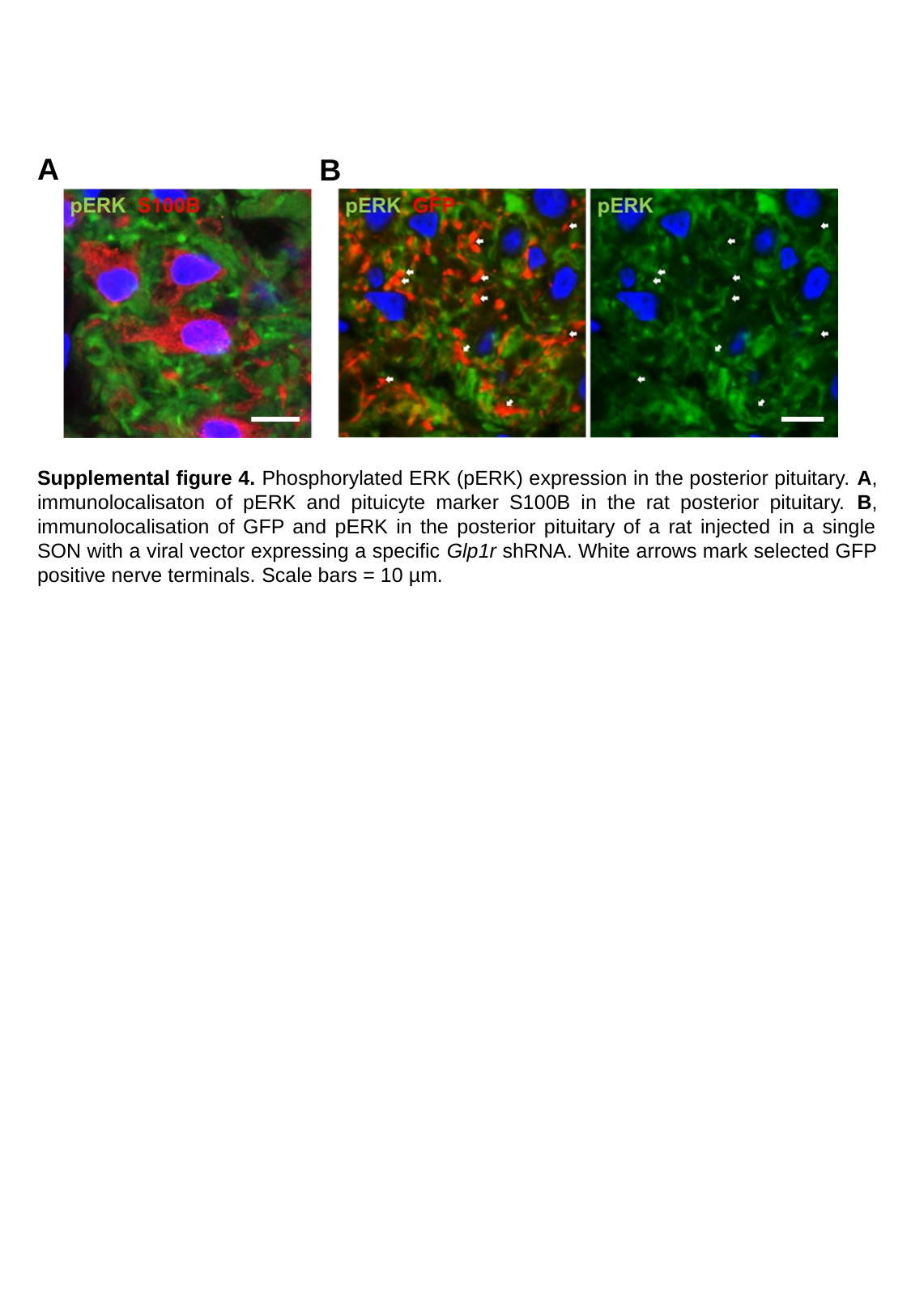

A
B
Supplemental figure 4. Phosphorylated ERK (pERK) expression in the posterior pituitary. A, immunolocalisaton of pERK and pituicyte marker S100B in the rat posterior pituitary. B, immunolocalisation of GFP and pERK in the posterior pituitary of a rat injected in a single SON with a viral vector expressing a specific Glp1r shRNA. White arrows mark selected GFP positive nerve terminals. Scale bars = 10 µm.

## Slide 5
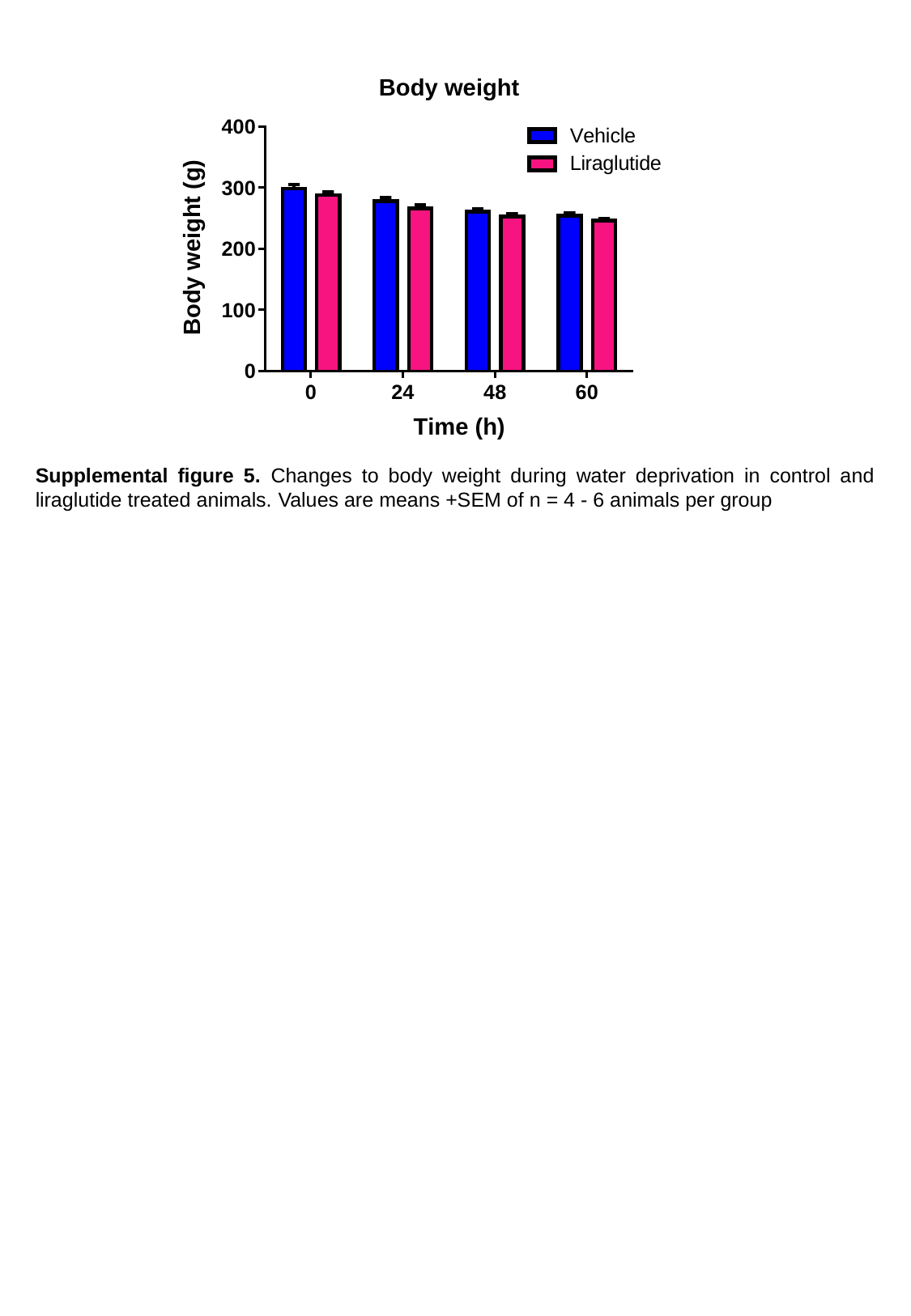

Supplemental figure 5. Changes to body weight during water deprivation in control and liraglutide treated animals. Values are means +SEM of n = 4 - 6 animals per group
