# Supplementary material for: Osmoadaptive GLP-1R signalling in hypothalamic neurones inhibits antidiuretic hormone synthesis and release
Source: Mol Metab. 2023 Feb 10;70:101692. doi: 10.1016/j.molmet.2023.101692 (PMC9969259; doi:10.1016/j.molmet.2023.101692)

Western blot lanes

Fig. 3B – Brain regions

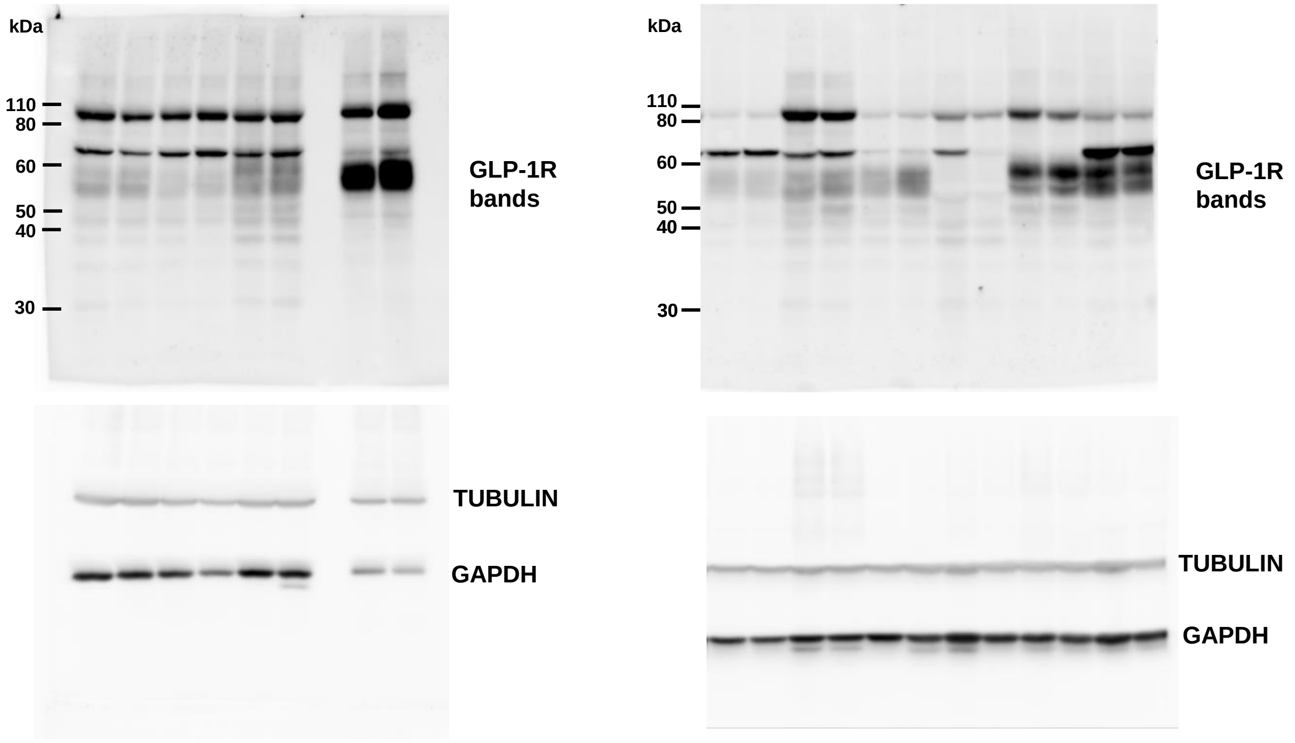

Fig. 3C – Control and 3 days water deprivation

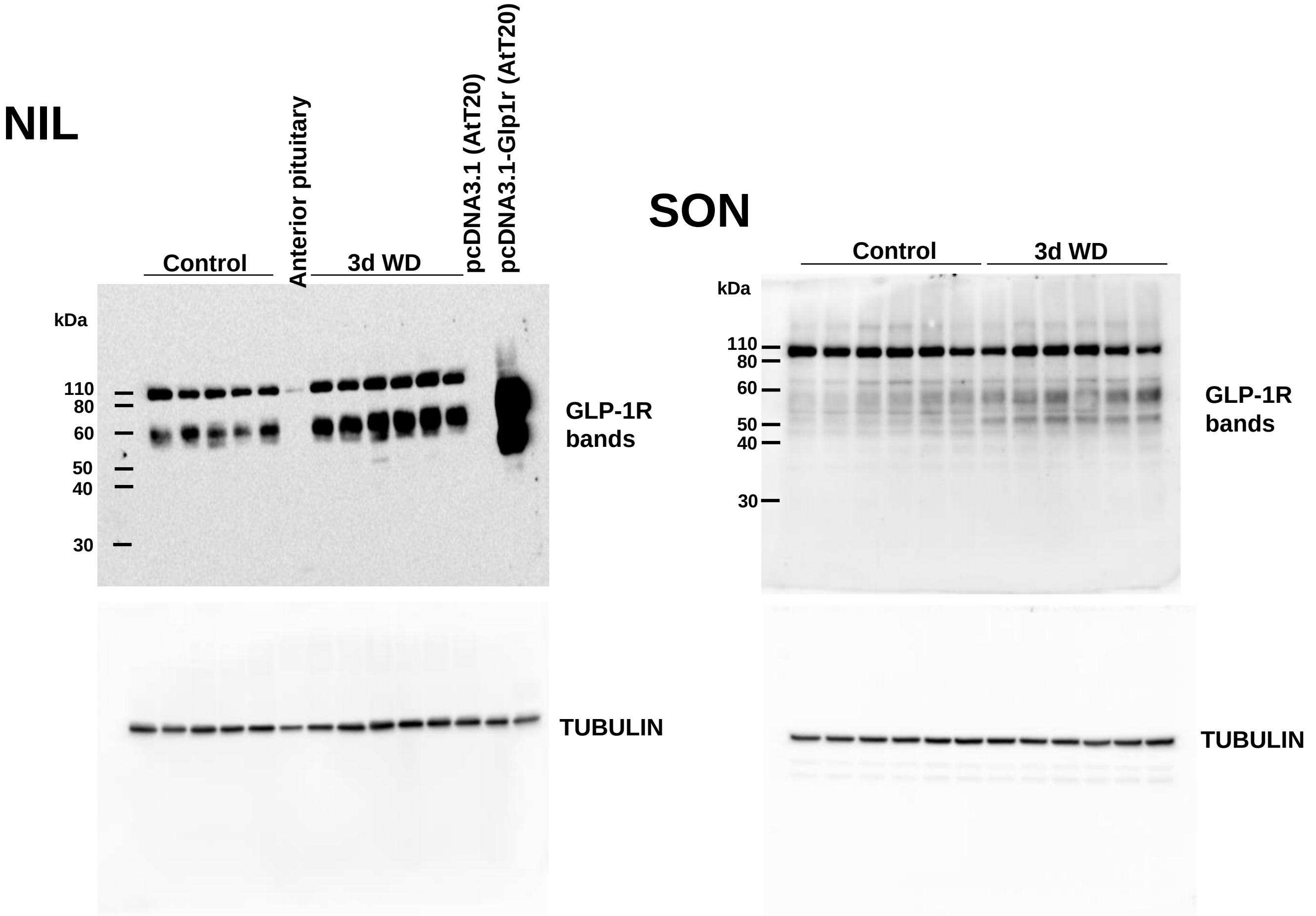

Fig. 5F – *Glp1r* knockdown SONs

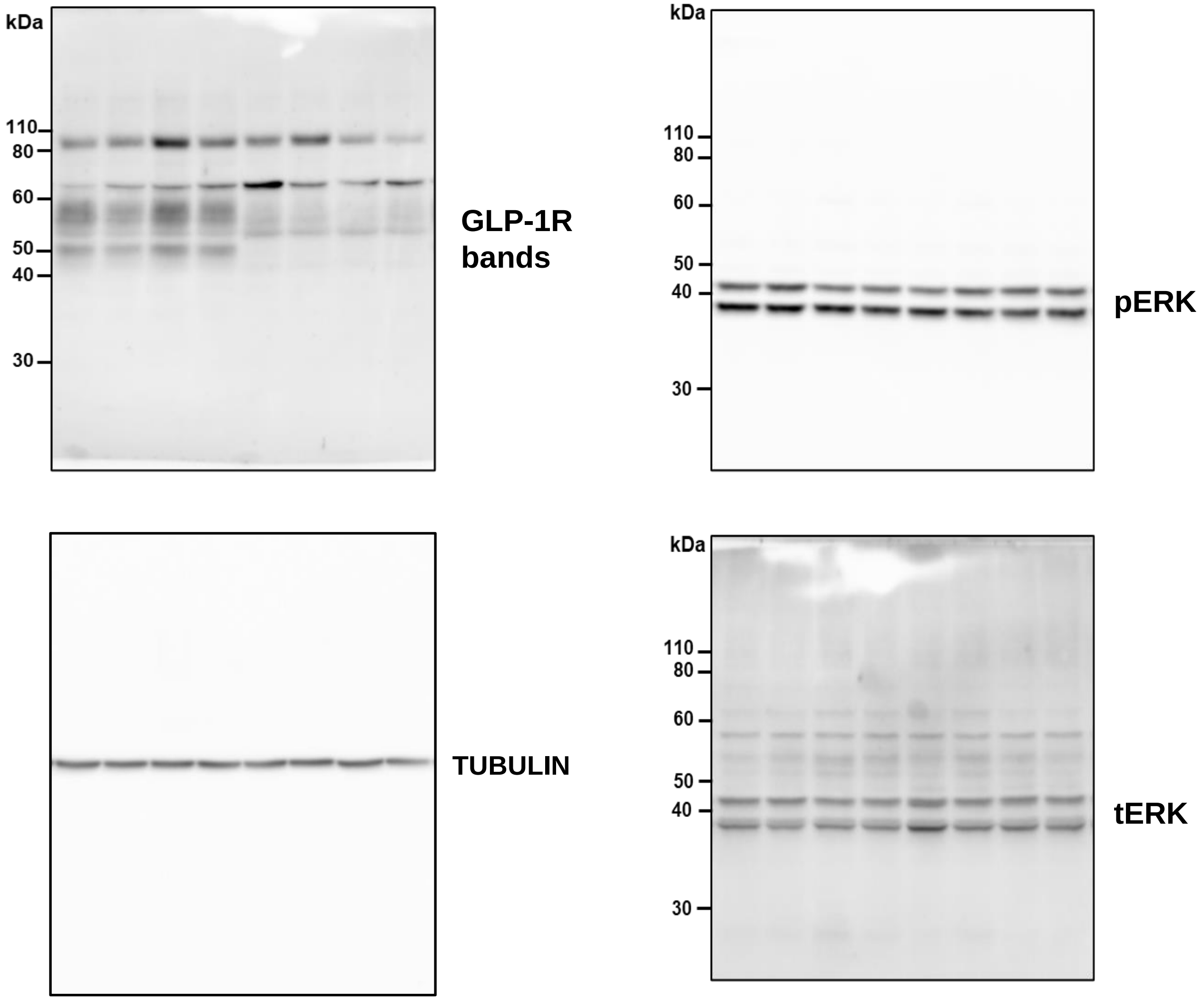

Fig. 8C – NILs treated for 30 minutes with liraglutide *in vivo*

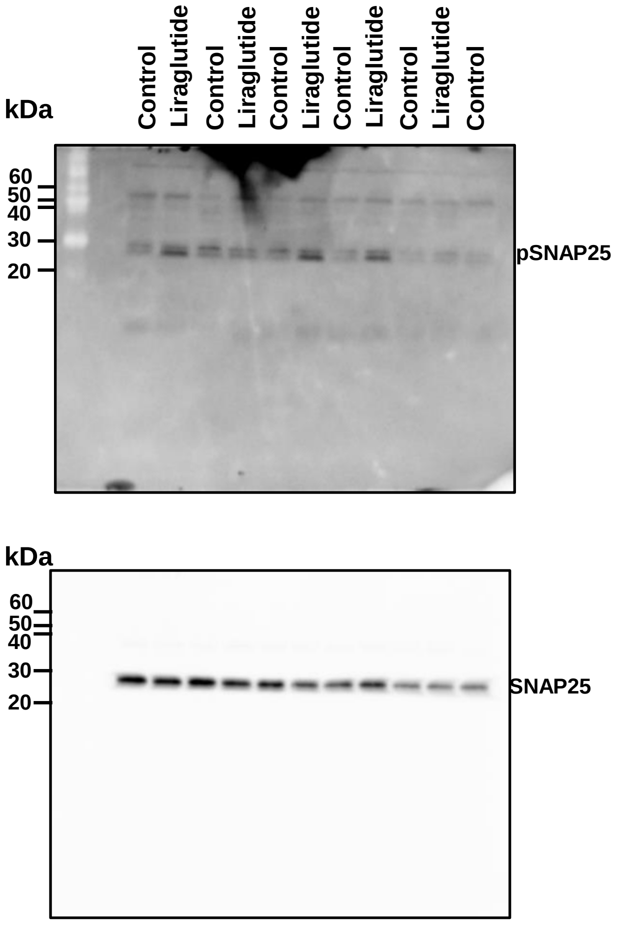

Fig. 8I – NILs treated with liraglutide for 30 minutes *ex vivo*

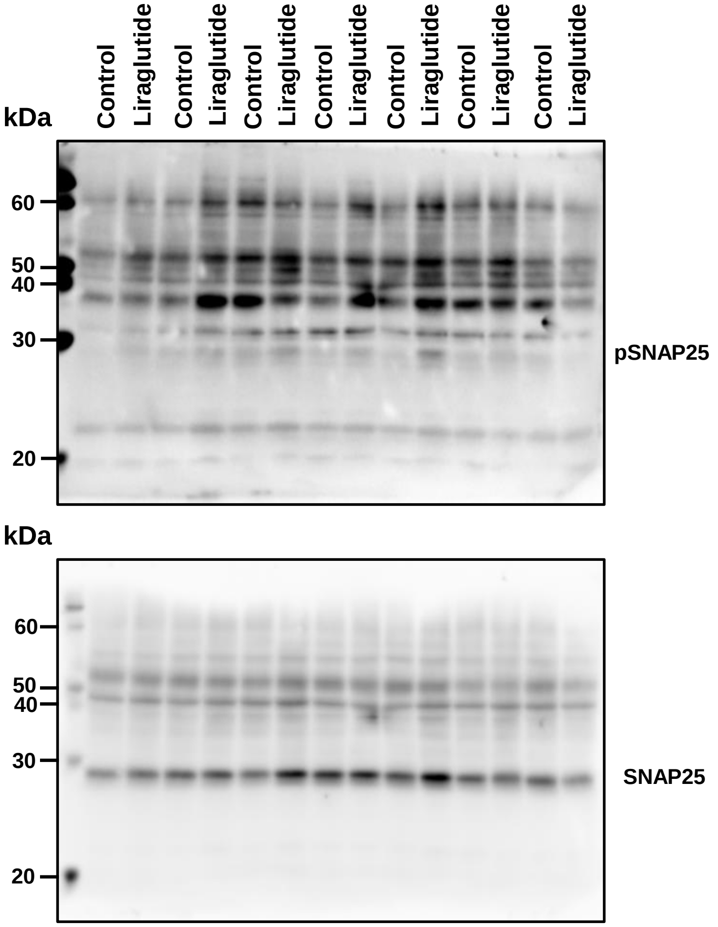

Supplement: Multimedia component 1 [file mmc1.zip › Supplemental files/Supplemental File 1.pdf]
