# Supplementary material for: Osmoadaptive GLP-1R signalling in hypothalamic neurones inhibits antidiuretic hormone synthesis and release
Source: Mol Metab. 2023 Feb 10;70:101692. doi: 10.1016/j.molmet.2023.101692 (PMC9969259; doi:10.1016/j.molmet.2023.101692)
Supplement: Multimedia component 1 [file mmc1.zip › Supplemental files/Supplemental Table 4.docx]

| Gene | Forward primer | Reverse primer |
| --- | --- | --- |
| **qRT-PCR primers** | | |
| *Creb3l1* | GAGACCTGGCCAGAGGATAC | GTCAGTGAGCAAGAGAACGC |
| *Creb3l1* | GCCAACAGGACCCTGCTCCA | AGTGCCAGTCTGTGTGGCCG |
| *Glp1r* | GTTCCGCTGCTGTTCGTTAT | GCAAGCGTATGATGAGCCAA |
| *Cckbr* | AACAAACCTGGTCCGTGCTA | CCATAGGCCACCGCAATAAC |
| *Oxtr* | CTACGTCACATGGATCACGC | TTGAAGCTGATGAGGCCGTA |
| *Gcg* | GGAGGAGAACGCCAGATCAT | CGGGAGTCCAGGTATTTGCT |
| *Fos* | AGCATGGGCTCCCCTGTCA | GAGACCAGAGTGGGCTGCA |
| *hnAvp* | GAGGCAAGAGGGCCACATC | CTCTCCTAGCCCATGACCCTT |
| *hnOxt* | TGAGCAGGAGGGGGCCTAGC | TGCAAGAGAAATGGGTCAGTGGC |
| *Avp* | TGCCTGCTACTTCCAGAACTGC | AGGGGAGACACTGTCTCAGCTC |
| *Oxt* | TGCCCCAGTCTTGCTTGCT | TCCAGGTCTAGCGCAGCCC |
| *tGfp* | AACACCCGCATCGAGAAGTA | GTGCCCATCACCTTGAAGTC |
| *Gapdh* | ATGACTCTACCCACGGCAAG | CTGGAAGATGGTGATGGGTT |
| *Rpl19* | GCGTCTGCAGCCATGAGTA | TGGCATTGGCGATTTCGTTG |
| *Sdha* | GGCGGGATTCCCACTAACTA | ACAGACCAGGCACAATCTGA |
| **Promoter primers** | | |
| *Glp1r-prom* | CGGGGTACCAGAAACCAGGCACCCCGCCCATACAT | CCGCTCGAGGGCGGGGCGCTCAGGACTGGGTCATA |
| ***Glp1r* shRNA** | | |
| *Glp1rsh1* | GCTCCTGTCGCTGTACATTAT | |
| *Glp1rsh2* | GGTGTTCCTGCTCATGCAATA | |
| *Glp1rsh3* | GCACGCATGAAGTCATCTTTG | |

**Primer table**

**Supplemental Table 1**. Oligonucleotides used in this study.
